# Discovery to Engineering of Mycotoxin Deoxynivalenol Degrading Enzymes Based on the Specialized Glyoxalase I

**DOI:** 10.1002/advs.202502914

**Published:** 2025-06-23

**Authors:** Seung Hee Lee, Song Lim Ham, Kyoungmi Oh, Hyojin Park, Young‐Seo Kang, Tae‐Joo Yang, Taekyung Kim, Jonghwan Kim, Gyu Sung Lee, Min‐Jeong Lee, Jin‐Byung Park, Chung Sub Kim, Nam Yoon Kim

**Affiliations:** ^1^ CJ BIO Research Institute CJ CheilJedang Suwon 16495 Republic of Korea; ^2^ Department of Biopharmaceutical Convergence Sungkyunkwan University Suwon 16419 Republic of Korea; ^3^ School of Pharmacy Sungkyunkwan University Suwon 16419 Republic of Korea; ^4^ Department of Food Science and Biotechnology Ewha Womans University Seoul 03760 Republic of Korea

**Keywords:** aromatase, biocatalyst, deoxynivalenol, detoxification, mycotoxin

## Abstract

Deoxynivalenol (DON) is a mycotoxin that is omnipresent in food and feed. Therefore, this study has focused on discovery, molecular characterization, and engineering of DON degrading enzymes, based on a DON isomerizing enzyme (e.g., the specialized glyoxalase I from *Gossypium raimondii* (Gr‐SPG)). A molecular phylogeny‐based sequence and structure analysis elucidated the evolutionary trajectory of the DON degrading enzymes. Ancestral sequence reconstruction led to the generation of thermostable evolutionary intermediates of SPG (e.g., Anc216). Molecular modeling and consensus protein design allowed to understand the structure and function relationships and also identify the key conserved mutations that influence catalytic activity and thermostability. Ultimately, a highly active and thermostable SPG (e.g., a quintuple mutant of Anc216 (Anc216_M5)) was constructed from a newly discovered extant SPG enzyme (OR9). The Anc216_M5 exhibited a $T_{50}^{10}$ of 68 °C, which is 16.3 °C higher than that of the wild‐type enzyme. Furthermore, the engineered enzyme showed 40% greater DON degrading activity than OR9, which is significantly higher than that of Gr‐SPG. Therefore, it is assumed that Anc216_M5 is promising as a DON‐detoxifying biocatalyst.

## Graphical abstract

Deoxynivalenol (DON), a prevalent food mycotoxin, is an important risk factor in global food safety. A molecular phylogeny‐guided engineering approach is devised to repurpose specialized glyoxalase I (SPG) as a DON deactivator. Engineered SPG variants demonstrate superior stability and are highly capable of converting DON into less toxic products, offering a promising solution for DON detoxification.

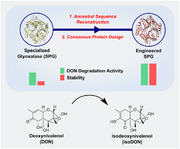

## 1 Introduction

**Scheme 1:**
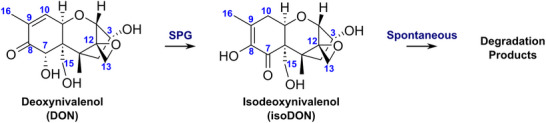
Transformation of deoxynivalenol (DON) by a specialized glyoxalase I (SPG). SPG‐catalyzed transformation of DON involves transfer of the C^9^‐C^10^ double bond to C^8^‐C^9^ and the C^8^ carbonyl to C^7^, forming isoDON, which undergoes spontaneous degradation.

Deoxynivalenol (DON) is a naturally occurring sesquiterpenoid metabolite that belongs to a class of mycotoxins known as trichothecenes (**Scheme** **1**). It is primarily produced by the *Fusarium* mold in cereal grains.^[^ ^1^, ^2^, ^3^, ^4^ ^]^ As one of the most prevalent natural contaminants in food and feed, DON not only causes significant economic losses in grain industry worldwide, but also poses a major threat to both human and animal health.^[^ ^5^, ^6^ ^]^ In humans, consumption of food contaminated with DON is associated with gastroenteritis, nausea, diarrhea, and vomiting.^[^ ^7^, ^8^, ^9^, ^10^ ^]^ In monogastric animals such as poultry and swine, exposure to high concentrations of DON causes acute symptoms such as intestinal lesions, immunosuppression, malaise, diarrhea, and emesis.^[^ ^11^, ^12^, ^13^, ^14^ ^]^ Therefore, DON is an important risk factor in global food and feed safety.^[^ ^15^, ^16^ ^]^

Previous strategies to reduce the levels of DON in contaminated crops have mainly focused on physical and chemical methods, involving heat and ozone. Although these methods achieved efficient degradation of DON, they generated highly complex mixtures of uncharacterized degradation products which could further contaminate raw food and feed materials.^[^ ^17^, ^18^ ^]^ In contrast, biodegradation approaches have been shown to be specific, environmentally amenable, and highly capable of attenuating the harmful effects of mycotoxins in food and feed. These include enzymatic and microbial detoxification,^[^ ^19^ ^]^ the latter of which has received much attention in DON detoxification efforts. Bacterial strains such as *Pelagibacterium halotolerans* ANSP101^[^ ^20^ ^]^ and *Devosia insulae* A16^[^ ^21^ ^]^ were found to be able to oxidize the C3‐OH to form 3‐keto‐DON, while *Paradevosia shaoguanensis* DDB001^[^ ^20^ ^]^ and *Nocardioides* WSN05‐2^[^ ^22^ ^]^ could epimerize DON to form 3‐*epi*‐DON through successive oxidation and reduction reactions (Scheme S1, Supporting Information). On the other hand the bacterial strains such as BBSH 797,^[^ ^23^ ^]^ *Bacillus* sp. LS100,^[^ ^24^ ^]^ *Desulfitobacterium* PGC‐3‐9^[^ ^25^ ^]^ and *Eggerthella* sp. DII‐9^[^ ^26^ ^]^ converted DON into a non‐toxic metabolite DOM‐1. Although the enzymes responsible for the transformations have been isolated and investigated in detail, their low stability and/or dependence on expensive cofactors makes their application in DON detoxification challenging.

Glyoxalase I (GLO1, EC 4.4.1.5) catalyzes the isomerization of hemithioacetal adducts, which are formed in a spontaneous reaction between a glutathionyl group and aldehydes (e.g., methylglyoxal). Interestingly, a specialized glyoxalase I (SPG), which is involved in the gossypol pathway (e.g., SPG from *Gossypium raimondii* (Gr‐SPG)), has been shown to convert DON and its derivatives to less toxic compounds (e.g., isoDON, iso‐3A‐DON, and iso‐15A‐DON).^[^ ^27^, ^28^ ^]^ The SPG appeared to lose the glutathione (GSH) cofactor binding site and undergo a functional transition to an enzyme catalyzing nickel‐dependent aromatization of α‐hydroxycarbonyl‐bearing substrates (e.g., DON).^[^ ^27^ ^]^

**Figure 1:**
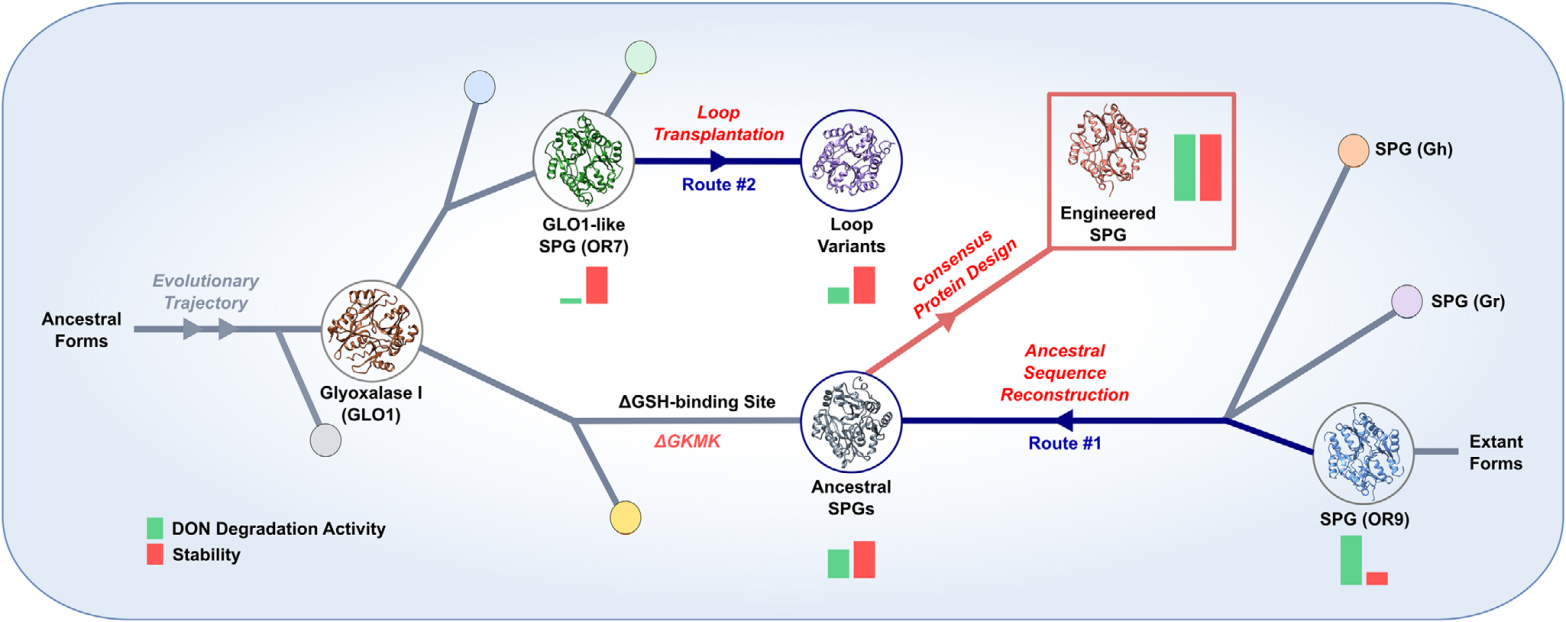
Phylogeny‐guided SPG engineering strategy. Bi‐directional engineering strategy (navy arrows) was designed based on the molecular phylogeny of SPG. It involves functional transition from glyoxalase I (GLO1) that detoxifies methylglyoxal to specialized glyoxalase I (SPG) that isomerizes cyclic sesquiterpenes containing α‐hydroxyketone moiety.

This study investigated a molecular phylogeny‐based bi‐directional evolution and engineering of GLO1 and SPG to create an efficient DON degrading enzyme (**Figure** **1**). Ancestral sequence reconstruction (ASR) and chimeragenesis led to a generation of evolutionarily distant variants of SPG. Molecular modeling and consensus protein design was used to identify the key conserved mutations among the extant SPG homologs, leading to a highly active and thermostable DON degrading enzyme. Overall, this study contributes to discovery, molecular understanding, and engineering of novel highly active DON degrading enzymes.

## 2 Results and Discussion

### 2.1 Discovery of Deoxynivalenol Degrading Enzymes

**Figure 2:**
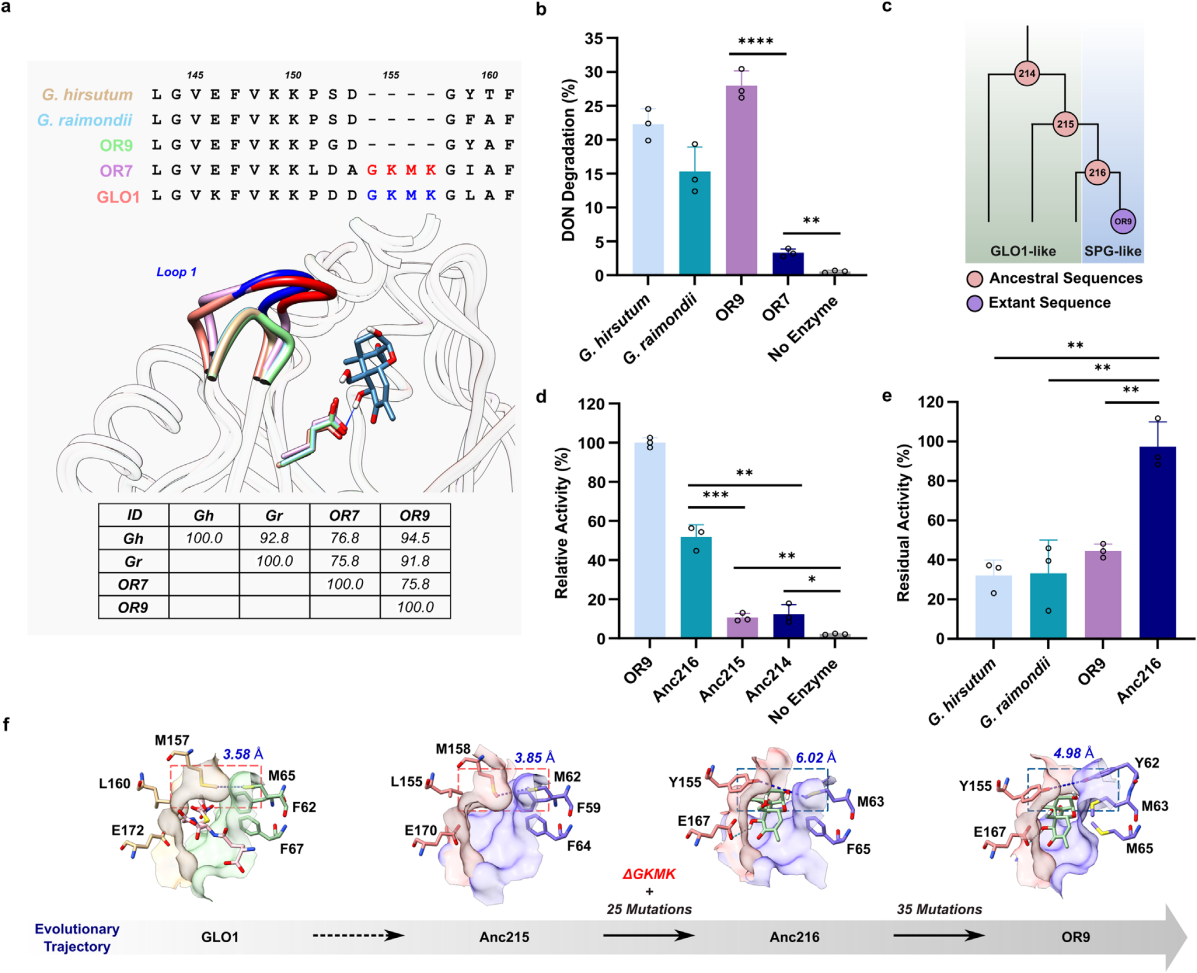
Identification of DON‐degrading SPG homologs and elucidation of the structural changes underlying their evolution via ancestral sequence reconstruction. a) Sequence alignment and modeling of SPG homologs and GLO1 (PDB: 1QIP) to show key structural deviations within Loop 1. The key hydrogen bond between C^7^‐OH of DON and Glu 167 residue, which is conserved among SPG homologs, was displayed in the model of the DON‐OR9 binding structure. b) Enzymatic degradation of DON catalyzed by SPG homologs. Degradation assays were conducted by incubating reaction mixtures containing 20 µg purified enzyme, 500 µM nickel (II) chloride, and 3.4 mm DON in 50 mm Tris‐Cl, pH 9.0 at 50 °C for 4 h (*n *= 3). c) Ancestral nodes reconstructed from OR9, of which Anc216 lacks GKMK motif (SPG‐like) whereas Anc215 and Anc214 contain GKMK motif (GLO1‐like). d) Relative DON degradation activities of ancestral forms of OR9 with respect to the activity of OR9 (specific activity: 5.47 mU mg^−1^) (*n *= 3). e) Residual DON degradation activities of SPG homologs and Anc216 after heat‐treatment of the purified enzymes at 40 °C for 20 h prior to enzyme assays. Percentage of DON degradation by samples pre‐incubation at 40 °C was considered 100% activity (*n *= 3). f) Structures of GLO1 (PDB: 1QIP) and the ancestors of OR9 modeled with AlphaFold 2 to show key structural changes within the active site‐lining residues that occur during the evolution of SPG. The distance shown in blue indicates the bottleneck distance in the substrate access tunnel. Statistical analysis was performed by a two‐tailed unpaired Student's *t*‐test. Data are presented as means, and error bars represent the standard deviation. ^*^ *p *< 0.05, ^**^ *p *< 0.01, ^***^ *p *< 0.001, ^****^ *p *< 0.0001; ns, no statistical significance.

An SPG from *Gossypium raimondii* (Gr‐SPG), which had previously shown DON degradation activities,^[^ ^27^ ^]^ was used as a template to discover new enzymes. A sequence similarity network (SSN) analysis led to a finding of 4 homologs (OR7, OR8, OR9, and OR11; Table S1, Supporting Information). The structure modeling of the homologs suggested that DON could bind to the active sites. For instance, the DON‐OR9 binding model showed a key hydrogen bond between C^7^‐OH and Glu 167 residue (Data S4, Supporting Information), which is conserved among SPG homologs (**Figure** **2**a). The structure models also indicated a notable difference in Loop 1, which was reported to shift toward the active site to facilitate ES complex formation during catalysis.^[^ ^29^ ^]^ In particular, the GKMK motif of Loop 1, which was known as a GSH‐binding motif,^[^ ^27^ ^]^ was present in OR7 (GLO1‐like), whereas disappeared in OR9 (SPG‐like). HPLC‐UV analyses of enzyme reaction mixtures revealed that OR9 exhibited high DON degradation activity while OR7 showed low activity (Figure 2b). The OR9 enzyme could degrade 28% of DON under the reaction conditions. This value was also markedly greater than that of other SPGs from the *Gossypium* genus under identical reaction conditions. For instance, Gr‐SPG and Gh‐SPG (SPG from *Gossypium harknessi* *i*), which have previously been shown to exhibit DON degradation activities,^[^ ^27^ ^]^ degraded only 15% and 22% of DON, respectively (Figure 2b). Taken together, the GKMK motif‐lacking SPG enzyme, OR9 is a promising novel DON degradation enzyme.

### 2.2 Ancestral Sequence Reconstruction (ASR) of the SPG Homolog OR9

The FireProt^ASR^ web server^[^ ^30^ ^]^ was used to reconstruct and predict ancestral sequences (Ancs) of the SPG homolog OR9 (Figures S3 and S4, Supporting Information). The ancestor nodes (Anc216, Anc215, and Anc214) were selected and examined for the DON degradation activities in vitro (Figure 2c,d). Among the resurrected ancestors, Anc216 exhibited the highest DON degradation activity, which corresponds to 52% of the OR9 activity. On the other hand, Anc215 and Anc214 showed 11% and 12% of the DON degradation activities (Figure 2d).

To compare the thermostabilities, residual DON degradation activities of the extant SPG homologs and Anc216 were measured after incubating the purified enzymes at 40 °C for 20 h. Remarkably, the residual activity of Anc216 was 97%, while Gr‐SPG, Gh‐SPG, and OR9 remained below 33%, 32%, and 45%, respectively (Figure 2e). These results indicate that through evolution, DON degradation activities of SPG's have increased at the cost of thermostabilities.

With an aim to understand the evolutionary trajectory, multiple sequence alignment of the evolutionary intermediates and OR9 was investigated. Notably, a marked difference was observed in Loop 1, which was reported to shift toward the active site during catalysis.^[^ ^29^ ^]^ The GKMK motif was present in Loop 1 of Anc214 and Anc215 (GLO1‐like), whereas disappeared in Anc216 (SPG‐like) (Figure S5, Supporting Information). The truncated Loop 1 in Anc216 appears to increase the bottleneck distance in the substrate access tunnel to the active site from 3.85 Å (Anc215) to 6.02 Å (Figure 2f, blue‐dotted box), resulting in an expanded substrate‐access tunnel and greater exposure of the active to the solvent. This change might improve the binding affinity for bulky cyclic substrates (e.g., DON), while discouraging the binding of linear substrates (e.g., methylglyoxal‐GSH adduct). Therefore, it was assumed that major structural changes contributing to the functional shift from GLO1 to SPG has probably occurred during evolution from Anc215 to Anc216.

### 2.3 Molecular Characterization of GLO1‐Like SPG Homolog OR7

**Figure 3:**
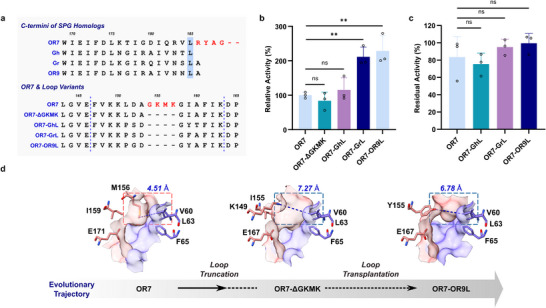
Loop transplantation to enhance the DON degradation activity of OR7. a) Sequence alignment of SPG homologs at C‐termini to show the presence of C‐terminal tail within OR7. Sequence alignment of loop transplanted variants to show the lack of GKMK motif as well as variations within residues 147–163, which compose Loop 1. b) Relative DON degradation activities of loop‐transplant variants with respect to OR7 (specific activity: 1.19 mU/mg) (*n *= 3). c) Residual DON degradation activities of OR7 and its loop variants after heat‐treatment of the purified enzymes at 40 °C for 20 h prior to enzyme assays (*n *= 3). d) Structures of OR7 and its loop variants modeled with AlphaFold 2 to show key structural changes within loop‐transplant variant OR7‐OR9L that confer increased DON degradation activity. The distance shown in blue indicates the bottleneck distance in the substrate access tunnel. Statistical analysis was performed by a two‐tailed unpaired Student's *t*‐test. Data are presented as means, and error bars represent the standard deviation. ^*^ *p *<0.05, ^**^ *p *<0.01, ^***^ *p *<0.001, ^****^ *p *<0.0001; ns, no statistical significance.

OR7 contains GSH‐binding GKMK motif within Loop 1, which resembles GLO1 (Figure 1). Previous studies have shown that deletion of GKMK motif within GLO1 abolished its GLO1 activity, while addition of GKMK motif into SPG partially restored GLO1 activity.^[^ ^27^ ^]^ Since our initial DON degradation assay (Figure 2b) indicated that OR7 exhibited basal activity for DON despite the presence of GKMK motif, we presumed that it might be possible to enhance its DON degradation activity by modifying Loop 1 to resemble that of the extant SPGs. To this end, a multiple sequence alignment of OR7 with extant SPGs was carried out. OR7 contains GKMK motif as well as C‐terminal tail that distinguishes itself from other SPG homologs (**Figure** **3**a, top). Interestingly, OR7 was expressed in a catalytically active form only after truncation of the C‐terminal tail after Leu185. Finally, catalytically active Loop 1 variants of OR7, which included GKMK deletion mutant and/or loop chimeras bearing Loop 1 from SPG homologs, were constructed.

The GKMK deletion mutant (OR7‐ΔGKMK) did not show a significant change in DON degrading activity (Figure 3b). However, the loop chimeras OR7‐GrL and OR7‐OR9L led to more than 2‐fold increases in activity. Molecular modeling suggested that the bottleneck distance in the substrate access tunnel in the GKMK deletion mutant was 7.27 Å, which is markedly longer than that of OR7 (4.51 Å) (Figure 3d). Transplantation of the entire Loop 1 from OR9 into OR7 also elongated the active site bottleneck distance, but shorter than that of the GKMK deletion mutant (6.78 Å).

The effect of loop modifications on the thermostabilities of OR7 variants was also examined. Interestingly, no significant differences in residual activities were observed when the loop variants were subjected to heat‐treatment for 20 h at 40 °C (Figure 3c). This result indicated that changes in Loop 1 structure had a marked impact on DON degrading activities but not on structural stabilities. In summary, not only the loss of GKMK motif but also mutations in Loop 1 that lead to fine‐tuning of the active site vicinity structure appeared to play a key role in a functional shift from GLO1 to SPG.

### 2.4 Engineering of a Highly Thermostable Ancestral SPG Variant

**Figure 4:**
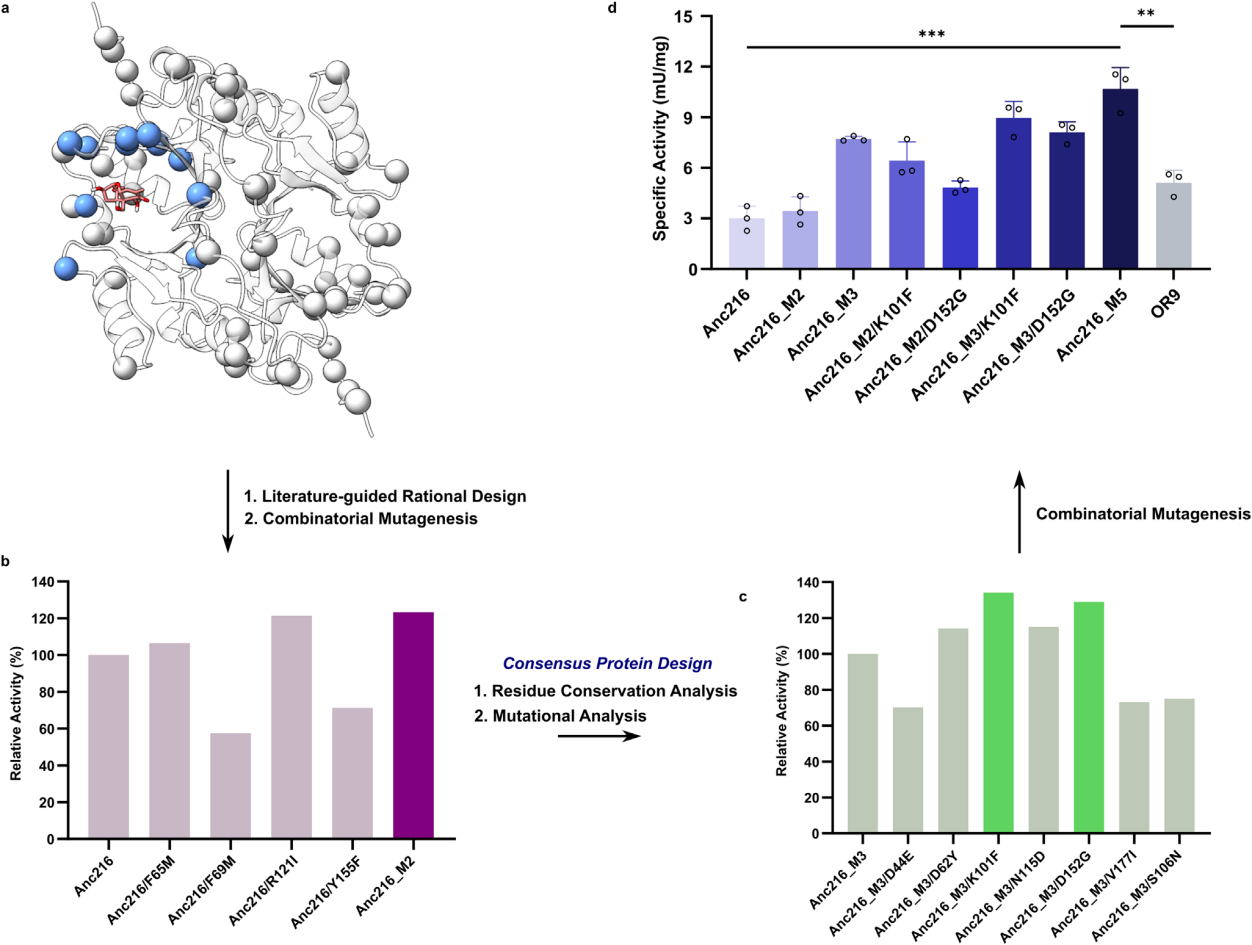
Consensus protein design. a) Comparison of the protein sequences to show the 35 mutations (spheres) that have occurred during the resurrection of Anc216 from OR9. The 11 mutations lining the active site (T121R, G152D, F101K, M65F, I28F, Y62D, I177V, L181V, V58I, M69F, N56K) are displayed in blue. b) 1st round of combinatorial mutagenesis involving 4 mutations (F65M, F69M, R121I, Y155F) selected via literature‐guided rational design, which yielded Anc216_M2 (F65M/R121I). c) 2nd round of combinatorial mutagenesis involving mutations selected through residue conservation analysis (Data S3, Supporting Information), which identified F28I as the key activity‐enhancing mutation in Anc216_M3 (Anc216_M2/F28I). Additional mutations, K101F and D152G, further improved the activity of Anc216_M3. d, 3rd round of combinatorial mutagenesis which yielded Anc216_M5 (F28I/F65M/K101F/R121I/D152G).

Anc216, an ancestor of OR9 showed very high thermal stability but low activity as compared to OR9 (Figure 2). Therefore, Anc216 was engineered to improve the DON degradation activity without losing the thermostability. Our strategy was to identify the key conserved mutations among the extant SPG homologs and to introduce them into Anc216 (Figure 1). Since stability‐determining mutations are often found at the surface, the mutations that are in proximity to the active site were focused on. Comparison of the protein sequences revealed that 35 mutations have occurred during the resurrection of Anc216 from OR9, among which we rationally identified 11 mutations lining the active site that may significantly impact substrate binding (T121R, G152D, F101K, M65F, I28F, Y62D, I177V, L181V, V58I, M69F, and N56K) (**Figure** **4**a). More specifically, our focus was on the mutations that may be further substituted to expand and reshape the active site to better accommodate the cyclic, compact structure of DON, thereby improving *K*_M_. Residue conservation analysis via multiple sequence alignment of the four DON‐degrading SPG homologs (validated in‐house) and Anc216 (Figure S6, Supporting Information) identified 22 mutations that are 100% conserved, and 14 mutations that are 75% conserved among the extant SPG homologs (Table S4, Supporting Information). Prior to screening these 36 mutations for improvement in catalytic activity, we first investigated a small set of rationally selected mutations around the active site (F65M, F69M, R121I, and Y155F),^[^ ^27^, ^28^ ^]^ following literature analysis. Notably, Anc216_F65M/R121I (Anc216_M2) exhibited 23% higher catalytic activity (Figure 4b) and 3.4‐fold greater residual DON degradation activity after heat treatment at 60 °C than Anc216 (Datas S1 and S2, Supporting Information).

Following the experimental verification of the remaining 34 single mutations (Data S3, Supporting Information), 7 single mutants demonstrated more than a 10% improvement in DON degradation activity. These mutants were F28I, D44E, D62Y, K101F, N115D, D152G, and V177I (Figure 4c). Among the seven mutations, five (K101F, D152G, D62Y, V177I, F28I) were among the 11 active site‐lining mutations we initially identified, with F28I exhibiting the highest improvement in activity (>157.9% of Anc216_M2). Combinatorial mutagenesis of the five active site mutations led to a number of interesting variants (Figure 4d). One of them was a quintuple mutant, Anc216_M5 (Anc216_ F28I/F65M/K101F/R121I/D152G), which has shown 3.6‐fold and 1.9‐fold greater specific activity toward DON degradation than that of Anc216 and OR9, respectively.

The kinetic study revealed that the *k*_cat_, *K*_M_, and *k*_cat_/*K*_M_ value of Anc216_M5 was 5.4 min^−1^, 51 mm, and 104 min^−1^ M^−1^ (**Table** **1**). Although the *K*_M_ value was higher than that of OR9, the *k*_cat_ was significantly greater, resulting in a 41% greater catalytic efficiency as compared to the OR9. This result indicated that Anc216_M5 is one of the most active DON degrading enzymes ever reported.

**Table 1:** Kinetic parameters of SPG variants.

Variant^a)^	*k*_cat_ [min^−1^]	*K*_M_ [mM]	*k*_cat_/*K*_M_ [min^−1^ M^−1^]	Fold Change
OR9	2.8 ± 0.7	38.1 ± 7.6	74 ± 23	1.0
Anc216	3.1 ± 1.0	82.7 ± 5.1	37 ± 12	0.5
Anc216_M5	5.4 ± 0.6	51.3 ± 9.8	104 ± 23	1.4

^a)^ Kinetic parameters were measured by using purified enzyme. All measurements were conducted in 50 mm Tris‐HCl buffer, pH 9.0, 50 °C. Detailed reaction conditions can be found in the Experimental section. *k*_cat_ and *K*_m_ were obtained by fitting the Michaelis‐Menten equation with GraphPad Prism v.10.0.

### 2.5 Structural Basis for Enhanced DON‐Degradation Activity

**Figure 5:**
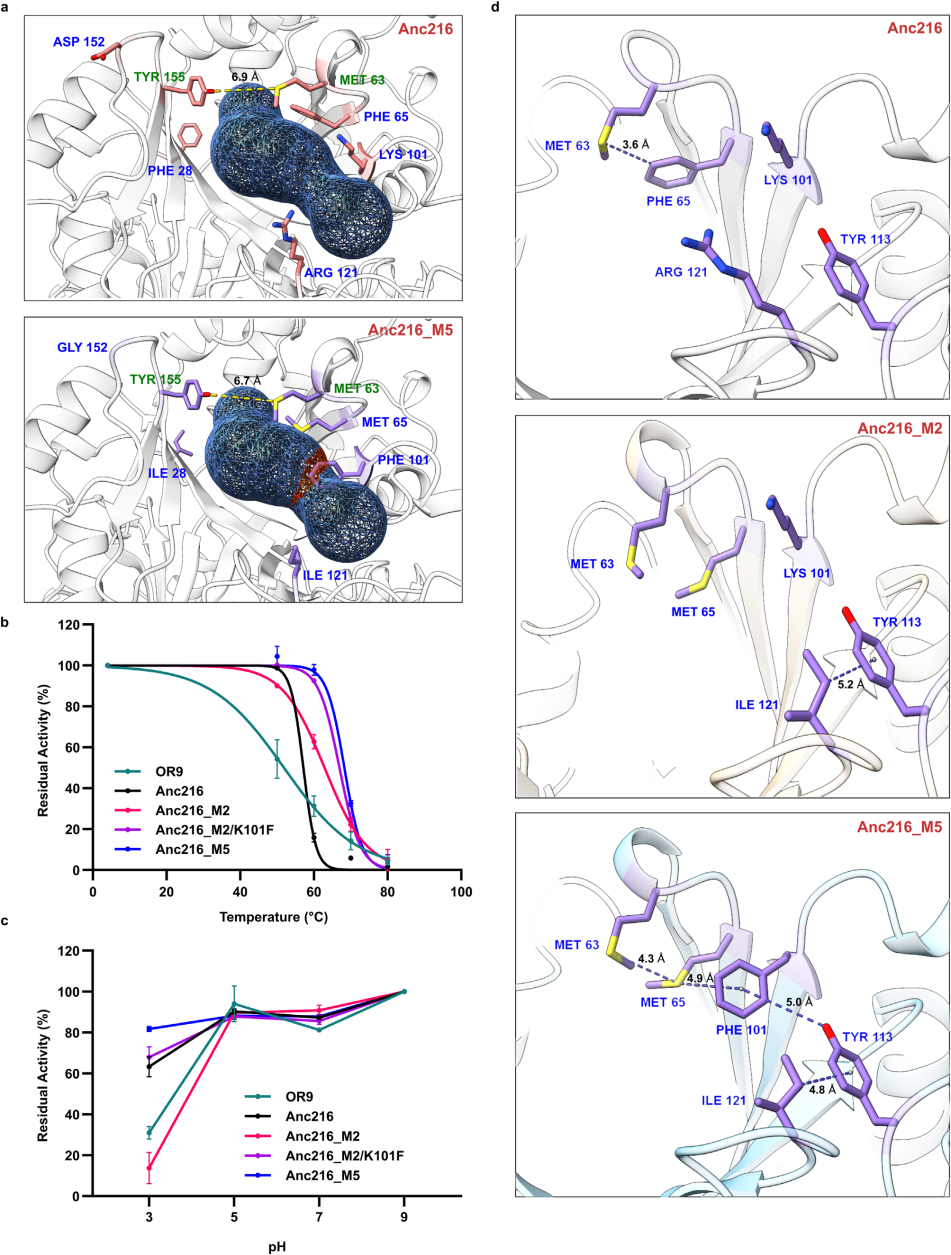
Structural basis for the enhanced activity and stability of Anc216_M5 variant. a) Key mutations selected from the residue conservation analysis of SPG homologs bearing high DON degradation capabilities (blue). Residues that determine the active site bottleneck are shown in green. b) Thermal deactivation profiles of OR9, Anc216, Anc216_M2, Anc216_M2/K101F, and Anc216_M5 (n = 3). c) pH stability profiles of OR9, Anc216, Anc216_M2, Anc216_M2/K101F, and Anc216_M5 (n = 3). d) Non‐covalent interactions in proximity to the active sites of Anc216, Anc216_M2, and Anc216_M5.

To understand the mutational effects on the DON degrading activities, the structure of Anc216_M5 was predicted. Interestingly, four of the 5 mutated residues (R121, K101, F65, and F28) line the substrate entrance tunnel while one residue (D152) is found in Loop 1 (**Figure** **5**a). The F65M mutation appeared to remove the bulk from Phe residue and add flexibility to the hydrophobic core within the active site. The tunnel analysis also indicated that the entrance of the tunnel of Anc216 is lined by positively charged and hydrophilic residues Arg121 and Lys101, which resembles GLO1. When these residues are mutated to Ile and Phe, respectively, they become neutral and hydrophobic, which may result in favorable interactions with DON. Additionally, D152G mutation, located on a flexible loop that presumably shifts toward the active site with the substrate binding,^[^ ^29^ ^]^ also removes negative charge, which may cause unfavorable interactions with *cis*‐enediolate intermediate. Taken together, the mutations D152G, R121I, and K101F presumably remove the unnecessary electrostatic interactions and remodel the substrate tunnel to better accommodate the hydrophobic and cyclic substrates such as DON. The smaller *K*_M_ value of Anc216_M5 than that of Anc216 (Table 1) also supported the hypothesis. Overall, the enzyme engineering to “re‐specialize” the evolutionarily distant Anc216 variant led to construction of Anc216_M5, which showed the markedly greater DON‐degradation activity, surpassing that of the extant parent SPG, OR9.

### 2.6 Stabilities of Anc216 Mutants Against Heat and Acid Stresses

The effects of activity‐enhancing mutations in Anc216 variants on thermal and acid stabilities have been investigated. The $T_{50}^{10}$ value of OR9 was 52.0 °C, whereas the $T_{50}^{10}$ values of Anc216_M2, Anc216_M2/K101F, and Anc216_M5 have increased gradually up to 68.3 °C (Figure 5b). Most interestingly, adding K101F to Anc216_M2 led to an increase of 4 °C in $T_{50}^{10}$ value (66.7 °C). This result indicated that the K101F mutation might play a critical role in the themal stability of Anc216_M5 variant. The structural analyses of Anc216, Anc216_M2, and Anc216_M5 suggested that the hydrogen bonding networks among the five residues (Met63, Met65, Phe101, Tyr113, Ile121) in the substrate‐binding cavity have been significantly strengthened by the mutations (i.e., F28I/F65M/K101F/R121I/D152G) (Figure 5d). Among them, the K101F mutation seemed to be critical for the stabilization of the substrate‐binding cavity via potential Met‐Met‐Aro‐Aro‐alkyl (M63‐M65‐F101‐Y113‐I121) interaction, where Met‐Aro interactions are reported to contribute 1‐1.5 kcal mol^−1^ to overall protein stability^63^. This is supported by our DeepDDG^64^ predictions of the Anc216_M5 mutations (Table S5, Supporting Information), which indicate that the K101F significantly contributes to the stabilization of the structure.

The acid stability of OR9 was also significantly enhanced by the mutations (Figure 5c). While the acid stabilities of OR9 and Anc216_M5 were comparable at a range of pH 5–9, the residual activities of OR9 and Anc216_M5 at pH 3 were 30.9% and 81.7%, respectively. The intermediate variants Anc216_M2 and Anc216_M2/K101F displayed the residual activities of 13.7% and 67.9%, respectively. The substantial increase of acid stability by the K101F mutation suggested that the K101F mutation made a key contribution on the acid stability of Anc216_M5. The K101F mutation might lead to an acid stability presumably through strengthening of the hydrophobic interactions around the active site (Figure 5d). To further understand the molecular basis of the high acid stability by Anc216_M5, we analyzed structural differences between the models of OR9 and Anc216_M5. As protein surface charges are often balanced by residues in proximity, which reinforce protein stability, it is plausible that improved charge uniformity within Anc216_M5 as well as increased hydrophobic interactions contribute to the stability. To rationalize improved acid resistance, we also determined the isoelectric points of OR9 and Anc216_M5, which were 4.79 and 5.10, respectively. As protein oligomers have higher tendency to dissociate at pH equal to their pI, in acidic conditions, we expect the lower pI of OR9 to favor its inactivation, which is consistent with our experimental outcome. Taken together, the strengthened non‐covalent interactions in Anc216_M5 appear to be responsible for its enhanced thermostability and acid stability, which are superior to those of other reported DON‐degrading enzymes (**Table** **2**).

**Table 2:** Stability Comparison of DON‐Degrading Enzymes.

Enzyme	Organism	pH stability	Thermostability	Refs.
*Yo*DDH^[^ ^31^ ^]^	*Youhaiella tibetensis*	N/A	>20% activity after heat treatment at 60 °C for 10 min.	Shi Y. et al. (2024)
DepA^[^ ^32^ ^]^	*Devosia mutans* 17‐2‐E‐8	>60% after treatment at pH 5 for 12 h	Loss of activity after heat treatment at 50 ^oC^ for 1 hr	Yang H. et al. (2022)
Fhb7_M10^[^ ^33^ ^]^	*Thinopyrum ponticum*	N/A	>50% activity after heat treatment at 50 °C for 5 min.	Yang J. et al. (2024)
SPG_Anc216_M5	*Gossypium* sp.	>80% after treatment at pH 3 for 1 h	>50% activity after heat treatment at 70 °C for 10 min.	This study

## 3 Conclusion

This study shed a light on evolutionary trajectory of DON degrading enzymes through molecular phylogeny‐based sequence and structure analysis. In addition, ancestral sequence reconstruction, chimeragenesis, and consensus protein design allowed to generate highly active DON degradation enzymes. A quintuple variant of Anc216 (Anc216_M5), which had been resurrected from OR9 enzyme, exhibited a 1.4‐ and 2.8‐fold greater catalytic efficiency than that of OR9 and Anc216, respectively. Furthermore, the Anc216_M5 showed a $T_{50}^{10}$ of 68 °C, which is 16.3 °C higher than that of OR9. Overall, this study contributes not only to the molecular understanding of DON degrading enzymes but also the construction of highly active DON degradation enzymes.

## 4 Experimental Section

### General Experimental Procedures

DON and Nickel(II) chloride were purchased from Sigma. HPLC‐grade acetonitrile (Honeywell Burdick and Jackson, USA) and water (Mallinckrodt Baker, USA) were used for HPLC analysis. For all statistical analysis and curve fitting, GraphPad Prism 10 was used. To determine statistical significance, two‐tailed t‐test and two‐way ANOVA were performed.

### SSN Construction and Homolog Identification

The SSN of SPG was generated via Sequence BLAST function of the EFI‐EST webserver (https://efi.igb.illinois.edu/efi‐est/) with the protein sequence of SPG from *Gossypium raimondii* as the input sequence. Default parameters were used in the construction of SSN, which was visualized with Cytoscape 3.9.1.^[^ ^34^ ^]^ Selected homologous protein sequences were codon‐optimized for expression in *E. coli*, and synthesized by Cosmo Genetech (Seoul, Korea). Sequence alignment of protein sequences was depicted as a graphical illustration using ESPript3.^[^ ^35^ ^]^ Pairwise identity and similarity of the sequences were calculated via Clustal Omega.^[^ ^36^ ^]^

### Ancestral Sequence Reconstruction

The ancestor sequences of SPG OR9 were predicted through the FireProt‐ASR webserver^[^ ^38^ ^]^ (https://loschmidt.chemi.muni.cz/fireprotasr/) via one‐step calculation. After prediction, the resulting phylogenetic tree was visualized using the iTOL v6 (https://itol.embl.de/)^[^ ^37^ ^]^ and three ancestor nodes (216, 215, and 214) were selected for gene synthesis (Cosmo Genetech, Korea).

### Plasmid Construction and Mutagenesis

To obtain N‐terminally His_6_‐tagged proteins for in vitro assays, individual genes were synthesized and cloned into pET28a expression vector (Novagen) between the NdeI and NotI sites. Site‐directed mutants were generated by the QuikChange mutagenesis method with the corresponding pairs of primers (Table S3, Supporting Information) synthesized by Bionics (Seoul, Korea). All mutations were validated by sequencing (Bionics, Korea).

### Expression and Purification of SPG and Its Variants

N‐terminally His_6_‐tagged SPG and its variants were expressed in *E*. *coli* BL21(DE3) by transforming pET28a‐SPG constructs into BL21(DE3), growing to an OD600 of 0.6 at 37 °C, and then inducing with isopropyl *β*‐D‐1‐thiogalactopyranoside (1 mm) prior to further cultivating overnight under aerobic conditions at 250 rpm and 25 °C. Cells were lysed by sonication, and protein was purified from cell lysate via affinity chromatography (Ni‐NTA agarose resin, Qiagen). Purified proteins were collected in three fractions which were combined and concentrated by using an Amicon Ultra‐15 centrifugal filter unit with 10 kDa cutoff, and buffer‐exchanged with SPG buffer (50 mm Tris‐HCl, pH 7.4, 150 mm NaCl) for storage at 4 °C. Glycerol stocks were prepared for storage at −20 °C.

DON degradation assay. In a typical DON degradation assay, DON (3.4 mm) was treated with SPG (20 µg) in Tris‐HCl (pH 9.0) supplemented with NiCl2 (500 µm) in a total volume of 50 µL for 4 h at 50 °C. The reactions were directly diluted 100‐fold in water prior to HPLC‐UV analysis. HPLC‐UV analysis was performed with an Agilent 1260 Infinity II HPLC System equipped with a UV detector. A C18 Inertsil ODS‐3 5 µm analytical column, 4.6 mm x 250 mm (GL Sciences, Japan), was used and column temperature was kept at 30 °C. The isocratic mobile phase consisted of water‐acetonitrile (80:20) with a flow‐rate of 1 mL min^−1^. and a run time of 20 min.

To construct thermal deactivation profiles of SPG variants, each variant (50 µL, 4 mg mL^−1^) was pre‐incubated for 10 min at temperatures of 50, 60, 70, and 80 °C and cooled down for 2 h on ice prior to enzyme reactions. Residual activities were calculated by conducting DON degradation assays with heat‐treated variants and determining the amount of DON remaining via HPLC. The untreated enzyme was used as the control.

To determine the acid stabilities of SPG variants, each variant (50 µL, 4 mg mL^−1^) was pre‐incubated for 1 h at 37 °C in the following buffers prior to enzyme reactions: Glycine‐HCl (50 mm, pH 3.0), Sodium Acetate (50 mm, pH 5.0), Tris‐HCl (50 mm, pH 7.0), Tris‐HCl (50 mm, pH 9.0). Residual activities were calculated by conducting DON degradation assays with acid‐treated variants and determining the amount of DON remaining via HPLC. The pH optimum was treated as 100% activity.

### Specific Activity Calculation

One unit of enzyme activity was defined as the amount of enzyme that consumed 1 µmol of DON per minute in pH 9.0 and 50 °C. Specific activities of SPG variants were calculated via determination of the amount of DON consumed through HPLC analyses. For example, to calculate the specific activity for a variant (20 µg) that consumed 30% of DON (3.4 mm) in 4 h, the following equation was employed:

(1) $$\begin{array}{ccc} \mathrm{Enzyme}\;\mathrm{activity} & = & \frac{3.4\times 10^{-3}\times 50\times 10^{-6}\times 0.3\times 10^{6}\;\mu mol}{4\times 60\;min\times (20\;\mu g)}\\ & & \times \;1000\;\mu g\;mg-1=0.01063\;U\;\mathrm{mg}-1\\ & & =10.63\;\mathrm{mU}\;\mathrm{mg}-1 \end{array}$$

### Enzyme Kinetics

Enzyme kinetics of the SPG variants were determined using 20 µm of enzyme in a final volume of 50 µL. Reactions were conducted in 50 mm Tris‐HCl buffer (pH 9.0). DON concentrations ranged from 0.425 to 6.8 mm, with NiCl_2_ concentration of 500 µM. Reactions were incubated for 120 min. at 50 °C and stopped. The amount of DON consumed was quantified by HPLC analysis. Kinetic constants were calculated on the basis of Michaelis‐Menten kinetics using GraphPad Prism v.10.0.

### Molecular Modeling and Docking

Chemical structures of DON, 3‐acetyl‐DON, and 8,11‐Dihydroxy‐7‐keto‐δ‐cadinene were prepared and minimized using the Avogadro software.^[^ ^38^ ^]^ Crystal structure of SPG (PDB: 7VQ6) from *Gossypium hirsutum* was used in docking analyses of DON and 8,11‐dihydroxy‐7‐keto‐δ‐cadinene. AlphaFold 2 (multimer v3) was used to model the dimeric structures of SPG variants, which were visualized using UCSF Chimera 1.14^[^ ^39^ ^]^ and Chimera X.^[^ ^40^ ^]^ Docking was performed using Autodock Vina.^[^ ^41^ ^]^

### Active Site Volume Determination

Volumes of SPG active sites within AlphaFold 2 models of SPG variants were determined using CASTp (computed atlas of surface topography of proteins) analysis webserver.^[^ ^42^ ^]^ Volumes of the two corresponding active sites within SPG dimers were averaged for comparison.

### Non‐Covalent Interactions Analysis

Total number of salt bridges and hydrogen bonds within AlphaFold 2 models of SPG variants were predicted using ProteinTools webserver.^[^ ^43^ ^]^

### Substrate Tunnel Analysis

Substrate tunnels were analyzed by MOLEonline webserver^[^ ^44^ ^]^ with the following parameters: interior threshold 1.1 Å, bottleneck tolerance 3 Å, bottleneck radius 1,2 Å, probe radius 5 Å, surface cover radius 10 Å. Tunnels were visualized by Chimera X.

## Conflict of Interest

The authors declare no conflict of interest.

## Author Contributions

S.H.L., J.‐B.P., and N.Y.K. performed conceptualization. S.H.L., S.L.H., K.O., T.K., J.K., and G.S.L. performed data curation. S.H.L., K.O., H.P., T.‐J.Y., T.K., and N.Y.K. performed methodology. S.H.L., K.O., H.P., T.‐J.Y., Y.S.K., J.K., G.S.L., M.‐J.L., C.S.K., and N.Y.K. performed validation.S.H.L., S.L.H., K.O., N.Y.K. performed writing‐original draft. S.L.H. and K.O. performed formal analysis. T.‐J.Y., T.K., J.K., G.S.L., J‐B.P., C.S.K, and N.Y.K. performed writing‐review and editing. J.‐B.P., C.S.K., and N.Y.K. performed supervision. C.S.K. validated funding acquisition. N.Y.K. performed visualization.

## Supporting information



Supporting Information

## Data Availability

The data that support the findings of this study are available in the supplementary material of this article.
