# Supplementary material for: Discovery to Engineering of Mycotoxin Deoxynivalenol Degrading Enzymes Based on the Specialized Glyoxalase I
Source: Adv Sci (Weinh). 2025 Jun 23;12(29):e02914. doi: 10.1002/advs.202502914 (PMC12362738; doi:10.1002/advs.202502914)
Supplement: Supplementary file 1 — Supporting Information [file ADVS-12-e02914-s001.docx]

*Supporting Information*

*Discovery to Engineering of Mycotoxin Deoxynivalenol Degrading Enzymes*

*Based on the Specialized Glyoxalase I*

Seung Hee Lee,^[a]^ Song Lim Ham,^[b,c]^ Kyoungmi Oh,^[a]^ Hyojin Park,^[a]^ Young-Seo Kang,^[a]^ Tae-Joo Yang,^[a]^ Taekyung Kim,^[a]^ Jonghwan Kim,^[b,c]^ Gyu Sung Lee,^[b,c]^ Min-Jeong Lee,^[d]^ Jin-Byung Park,^[d]^ Chung Sub Kim,*^[b,c]^ Nam Yoon Kim*^[a]^

aCJ BIO Research Institute, CJ CheilJedang, Suwon 16495, Republic of Korea

bDepartment of Biopharmaceutical Convergence, Sungkyunkwan University, Suwon 16419, Republic of Korea

cSchool of Pharmacy, Sungkyunkwan University, Suwon 16419, Republic of Korea

dDepartment of Food Science and Biotechnology, Ewha Womans University, Seoul 03760, Republic of Korea

**Supplementary Tables**

Supplementary Table 1. Amino acid sequences of SPG homologs identified in this study

Supplementary Table 2. Amino acid sequences of OR9 ancestral forms predicted in this study

Supplementary Table 3. List of primers used in site-directed mutagenesis

Supplementary Table 4. Residue conservation analysis of activity-validated SPG homologs

Supplementary Table 5. DeepDDG predictions of mutations in 216M5

**Supplementary Figures**

Supplementary Figure 1. CASTp analysis of the active sites for GLO1 and OR7

Supplementary Figure 2. Surface representation of DON-binding active sites

Supplementary Figure 3. Phylogenetic tree constructed via FireProt-ASR

Supplementary Figure 4. Ancestral nodes selected for gene synthesis

Supplementary Figure 5. Multiple sequence alignment of OR9 and its ancestors

Supplementary Figure 6. Multiple sequence alignment of SPG homologs exhibiting high DON-degradation activity

**Supplementary Schemes**

Supplementary Scheme 1. Enzymatic transformations of deoxynivalenol

**Supplementary Data**

Supplementary Data 1. Relative activities of 216M2 variants

Supplementary Data 2. Thermostabilities of 216M2 variants

Supplementary Data 3. DON degradation activities of the selected mutations from residue conservation analysis

Supplementary Data 4. Mutational studies on the catalytic residues of SPG

Supplementary Data 5. Enzyme kinetics analysis of OR9, Anc216, and 216M5

Supplementary Data 6. Spontaneous degradation of isoDON at an elevated temperature

Supplementary Data 7. LC/MS total ion count (TIC) chromatograms of SPG-DON reaction mixtures

Supplementary Data 8. Estimated model accuracy of the AlphaFold 2-predicted structures of OR9, Anc216, and 216M5

Supplementary Data 9. Comparison of the AlphaFold 2-predicted structures with AlphaFold 3-predicted structures

**Supplementary Tables**

**Supplementary Table 1. Amino acid sequences of SPG homologs**

| **Homolog** | **Origin** | **Sequence** |
| --- | --- | --- |
| **Gh** | *Gossypium hirsutum* | MGSMDSKESPANNPGLHTPPDEATKGYIMQQTMFRIKDPKRTLEFYSRVLGMSLLNKVDVPYMKMTLYMMGYEDVSSAPSDPVEKTIWTFGRPATMELTHFWGTENDPEFKGYHNGNSEPIGFGHIGITVDDMYKACERF ESLGVEFVKKPSDGYTFIKDPDGYWIEIFDLNGIRAIVNTL |
| **Gr** | *Gossypium raimondii* | MASLDSKESPANNPGLHFPPDEATKGYIMQQTMFRIKDPKRTLEFYSRVLGMTLLNKVDVPYMKMTLYMMGYEDVSSAPTDPVEKTIWTFGRPATMELTHFWGTENDPEFKGYHDGNSEPIGFGHIGLTVDDLYKACERFESLGVEFVKKPSDGFAFIKDPDGYWIEIFDLKGIRQIVNSLA |
| **OR7** | *Gossypium barbadense* | MASSDLKESAANNPGLHTTPDEATKDYIMQQTMYRIKDPKVSLDFYSRVLGMSLLKRVDVPELKFTLYFMGYEDVSKAPSDPYGRTVWTFGRAATIELTHNWGTESDPEFKGYHTGNSEPRGFGHIGITVDDVNKACERFERLGVEFVKKLDAGKMKGIAFIKDPDGYWIEIFDLKTIGDIQRVLRYAGIRISATCRHRFMLKEDFRISHREINSHGLMTIHLTISPPHCCFHFLNSKFVVSVNETRMACEGFISLSWFQSMVYIGRSQDGN |
| **OR8** | *Handroanthus impetiginosus* | MASSSLSAAFSCLRPAFQRPPLLSSLPTLFTLKPKVLNQPRRFVSTTTVMASKESPENNPGLQTSLDEATKGYFLQQTMLRVKDPKVSLDFYSRIMGMSLLKRLDFPELKFTLYFLGYEDTSSAPGNPVERTSWTFGQKAVLELTHNWGTETDPDFKGYHNGNSEPRGFGHIGITVDDVHKACKRFESLGVEFVKKPQDGKIKDIAFIKDPDGYWIEIFDTTTIAKTTASAAV |
| **OR9** | *Gossypium harknessii* | MGSLDSKESPANNPGLHSPPDEATKGYILQQTMFRIKDPKPALEFYSRVLGMSLLNKVDVPYMKMTLYMMGYEDVSSAPSDPVEKTIWTFGRPATMELTHFWGTENDPEFKGYHDGNSEPTGFGHIGITVDDMYKACERFESLGVEFVKKPGDGYAFIKDPDGYWIEIFDLNGIRAIVNNLA |
| **OR11** | *Hibiscus syriacus* | MASSGPKESAANNPGLQTPPEITKGYFMQQTCFRIKDPKVSLDFYSRILGMSLLKRIDVPDLKFTLYFMGYEDVSTAPSDPIDRTAWTFSKPATIELNHKWGTESDTEFKGYHTGNSEPLGFGHLGITVDDTFKACERFA RLGVEFVKTPEDGYAFIKDPDGYWIEIFDLVNIRNVVKGVA |

**Supplementary Table 2. Amino acid sequences of OR9 ancestors**

| **Ancestor** | **% ID (with respect to OR9)** | **Sequence** |
| --- | --- | --- |
| **216** | 80.77 | MASSGPKESAANNPGLQTPPDEATKGYFMQQTMFRIKDPKVSLDFYSRVLGMSLLKRIDVPDMKFTLYFMGYEDVSSAPSDPIERTAWTFGRPATIELTHKWGTESDPEFKGYHNGNSEPRGFGHIGITVDDTYKACERFERLGVEFVKKPDDGYAFIKDPDGYWIEIFDLKNIRNVVNGVA |
| **215** | 76.54 | MASESKESAANNPGLCSTPDEATKGYFMQQTMFRIKDPKVSLDFYSRVLGMSLLKRLDFPDMKFSLYFMGYEDPSSAPTDPTERTAWTFGRKATIELTHNWGTESDPEFKGYHNGNSEPRGFGHIGITVDDTYKACERFERLGVEFVKKPDDGKMKGLAFIKDPDGYWIEIFDLKNIRSVTSAAS |
| **214** | 75.98 | MASESKESAANNPGLCSTPDEATKGYFMQQTMFRIKDPKVSLDFYSRVLGMSLLKRLDFPEMKFSLYFMGYEDPASAPTDPTERTAWTFGRKATLELTHNWGTESDPEFKGYHNGNSEPRGFGHIGITVDDTYKACERFERLGVEFVKKPDDGKMKGLAFIKDPDGYWIEIFDLKNIRSVTSAAS |

**Supplementary Table 3. List of primers used in site-directed mutagenesis**

| **Mutation** | **Sequence** |
| --- | --- |
| F65M-F | CCCGGATATGAAGatgACGTTATATTTTATGGGATATGAAG |
| F65M-R | ACGTcatCTTCATATCCGGGACGTCAATTCTTTTCAAAAG |
| R121I-F | atcGGTTTCGGGCACATAGGCATCACGGTCGATGACACTTAC |
| R121I-R | CCATAATGGCAATTCTGAACCAatcGGTTTCGGGCACATAGG |
| F28I-F | GGCCACTAAGGGTTATattATGCAACAGACAATGTTTCGCAT |
| F28I-R | CATaatATAACCCTTAGTGGCCTCGTCAGGTGGAGTTTGCAAG |
| K101F-F | ATtttTGGGGTACCGAGAGCGACCCGGAGTTTAAAGGTTAC |
| K101F-R | GCTCTCGGTACCCCAaaaATGGGTCAATTCGATTGTCGC |
| D152G-F | AGAATTTGTGAAAAAGCCGggcGACGGCTATGCGTTTATTAAAGACCC |
| D152G-R | gccCGGCTTTTTCACAAATTCTACCCCAAGACGTTCGAATCTCTCACAC |

**Supplementary Table 4. Degree of residue conservation among activity-validated SPG homologs**


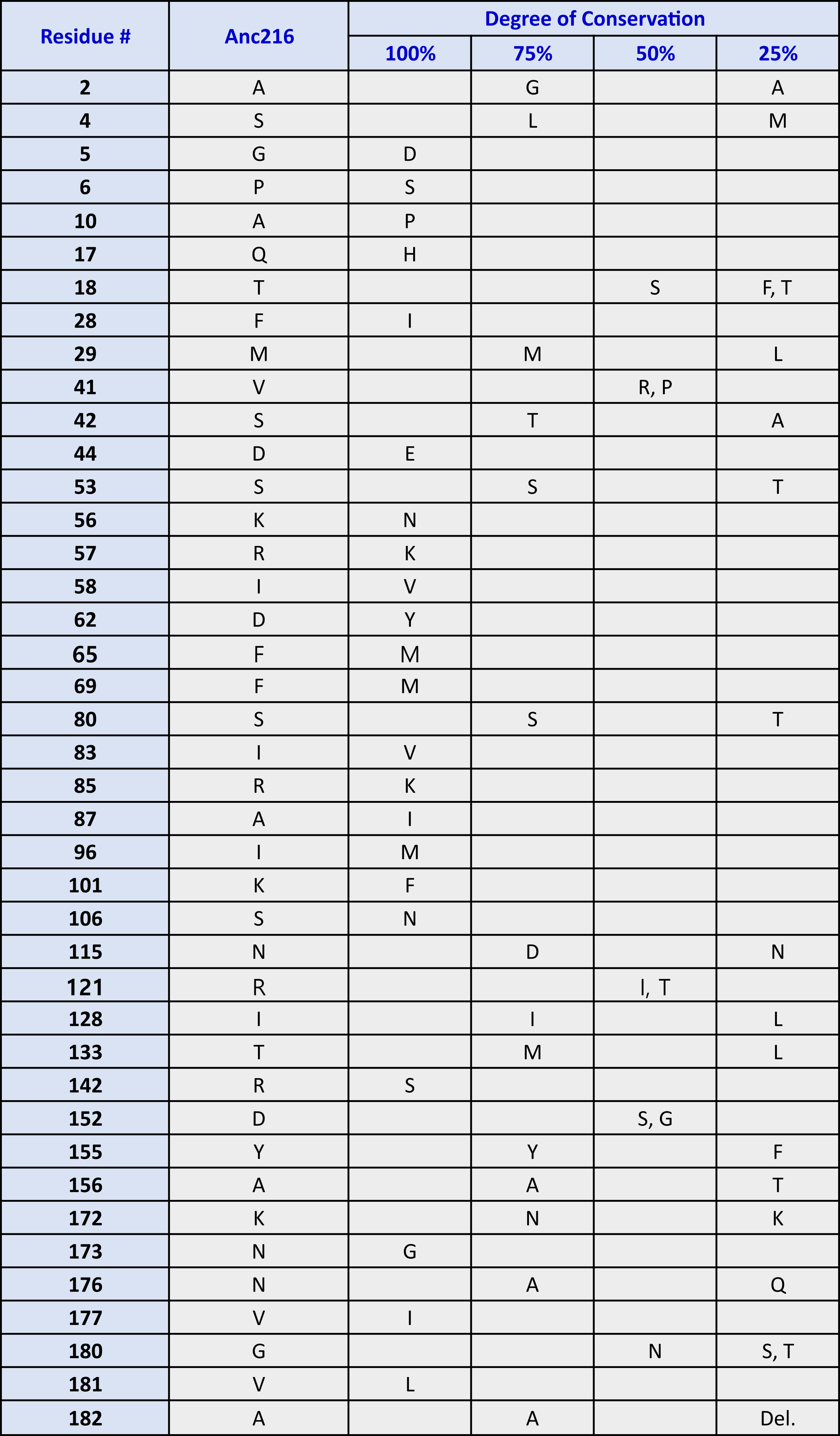


**Supplementary Table 5. DeepDDG predictions of mutations in 216M5**

| **Mutation** | **△△G (kcal/mol)** |
| --- | --- |
| F28I | -0.384 |
| F65M | -1.649 |
| K101F | 0.401 |
| R121I | -0.410 |
| D152G | -0.476 |

-Positive and negative △△G values correspond to stabilizing and de-stabilizing mutations, respectively.

**Supplementary Figures**


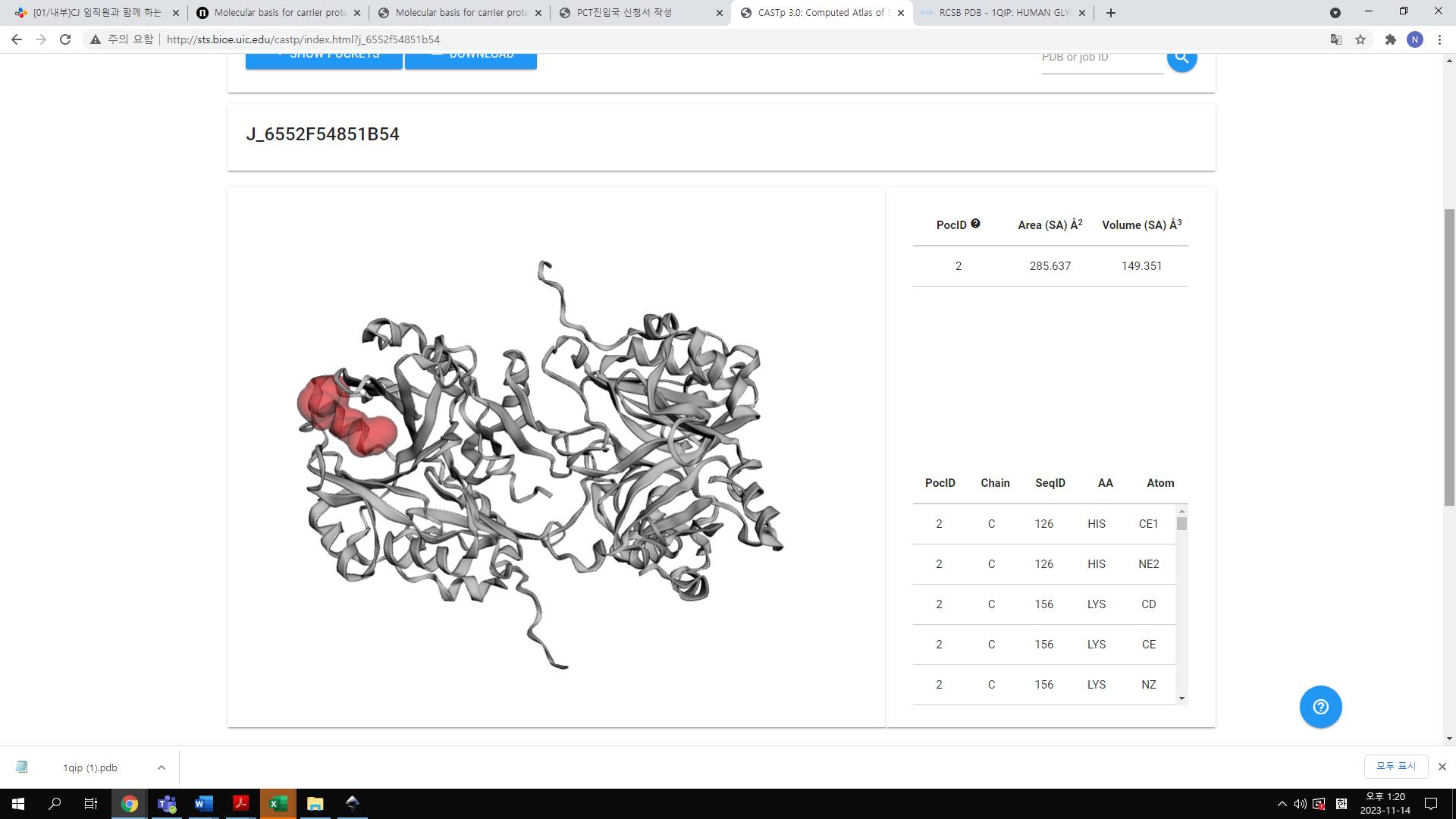

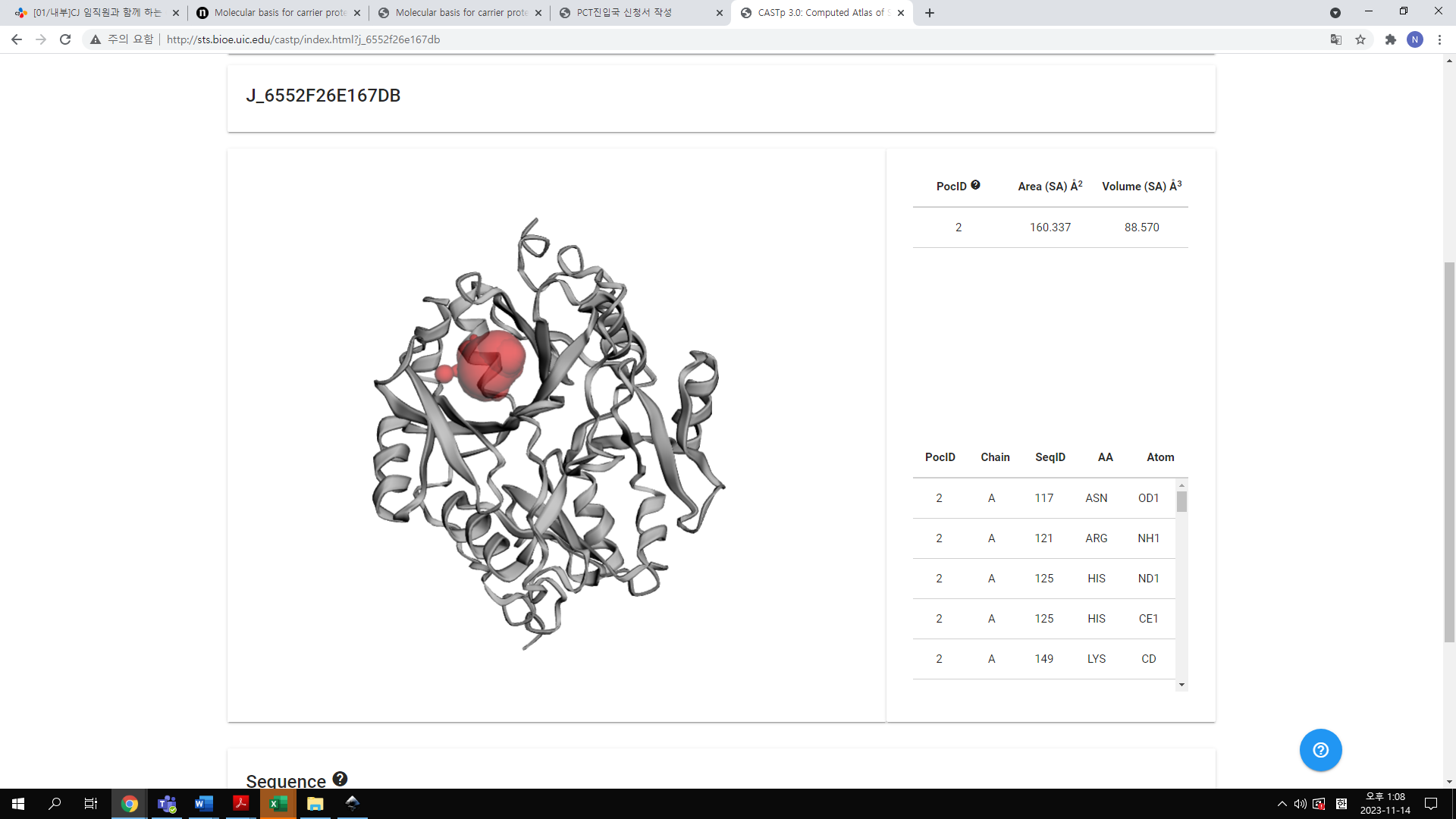


**Supplementary Figure 1. CASTp analysis of the active sites for GLO1 and OR7**. CASTp (computed atlas of surface topography of proteins) webserver was used to calculate the volumes of active sites for GLO1 (left, 149.35 Å3) and OR7 (right, 88.57 Å3).


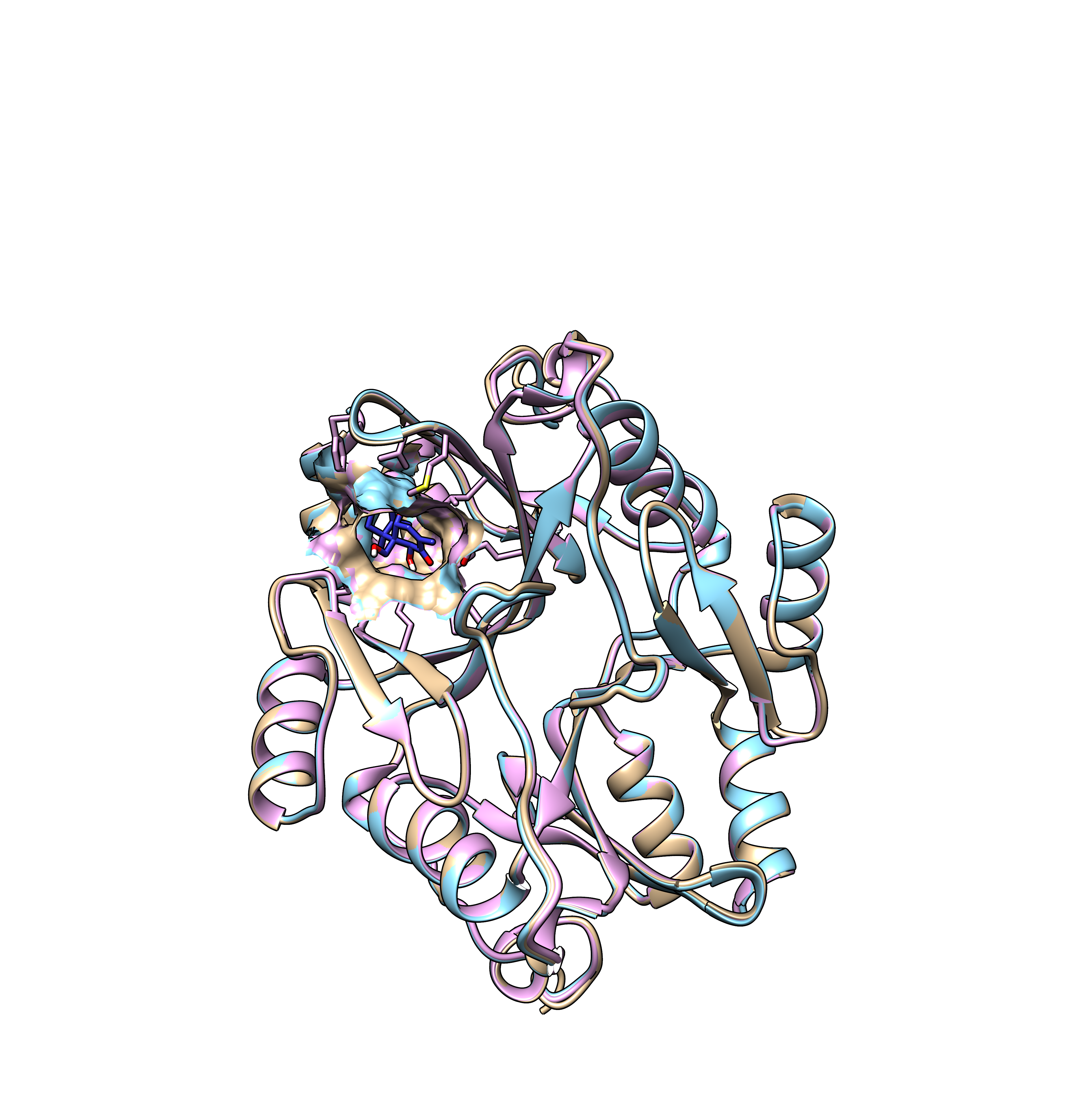

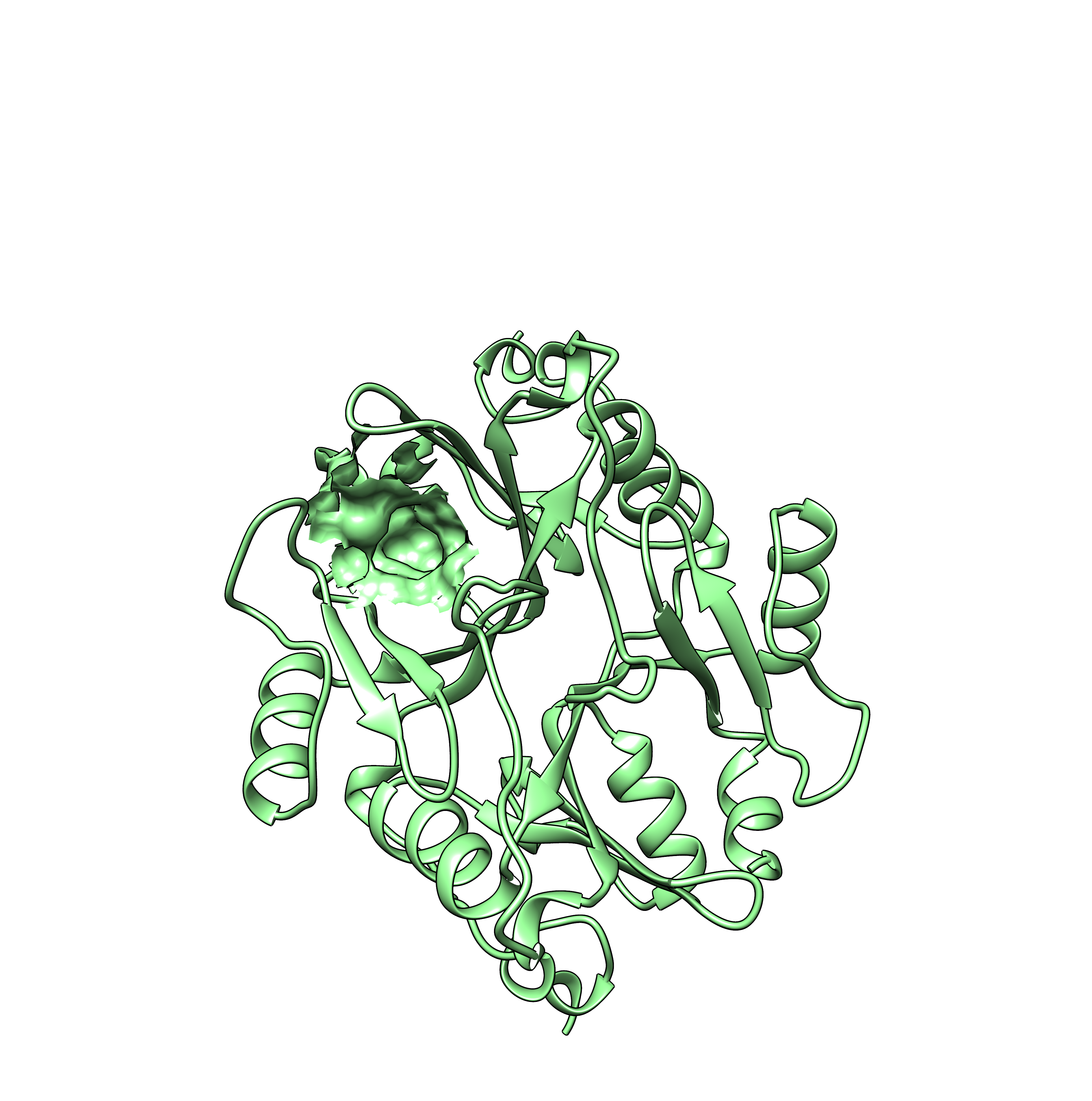


**Supplementary Figure 2. Surface representation of DON-binding active sites**. SPGs from *G. hirsutum*, *G. raimondii*, OR9, and OR7 were modeled using AlphaFold 2 (multimer v3), and AutoDock Vina was used to dock energy-minimized DON into OR9. Superimposition of GKMK-lacking homologs (*G.hirsutum* (gold), *G. raimondii* (blue), OR9 (purple)) show broadened active site compared to that of OR7 (green) which possess GSH-binding GKMK motif.


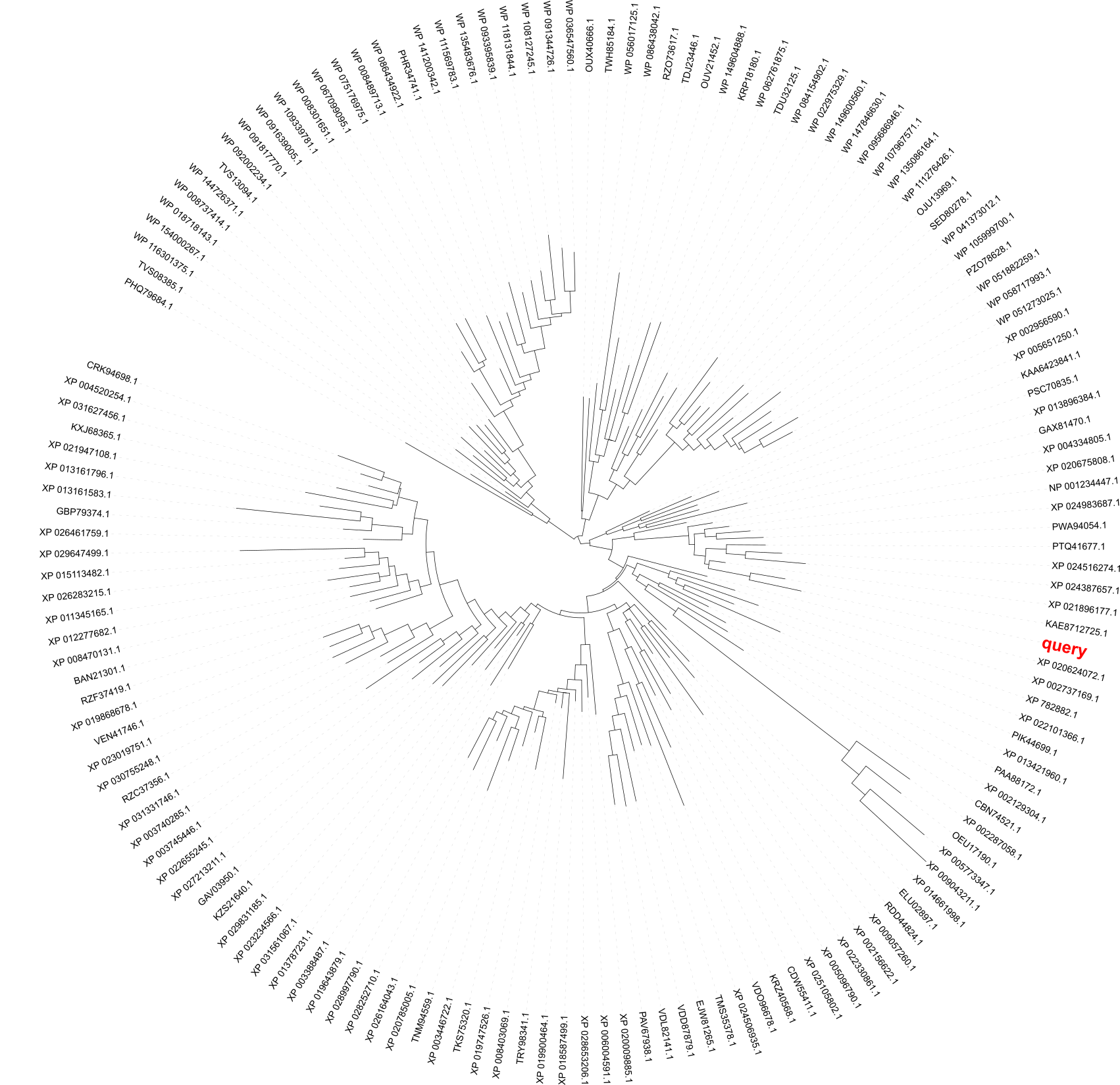


**Supplementary Figure 3. Phylogenetic tree constructed via FireProt-ASR.** Phylogenetic tree was constructed using OR9 protein sequence as an input and visualized via IQ-TREE webserver.


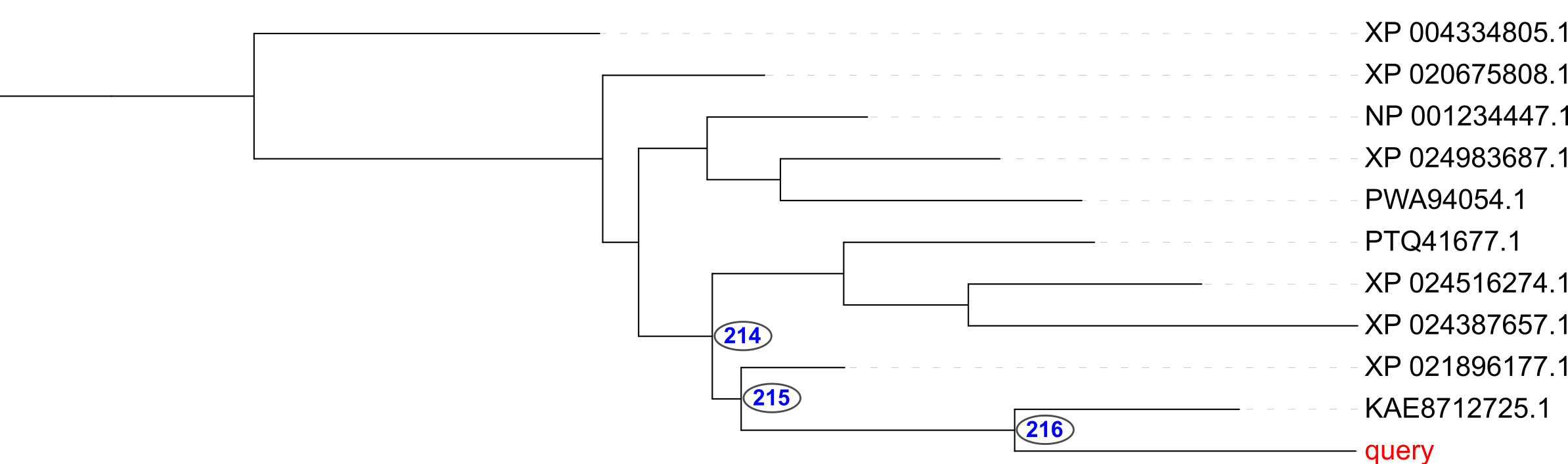


**Supplementary Figure 4. Ancestral nodes selected for gene synthesis.** Ancestor nodes 216, 215, and 214 were selected for gene synthesis and expression.

**
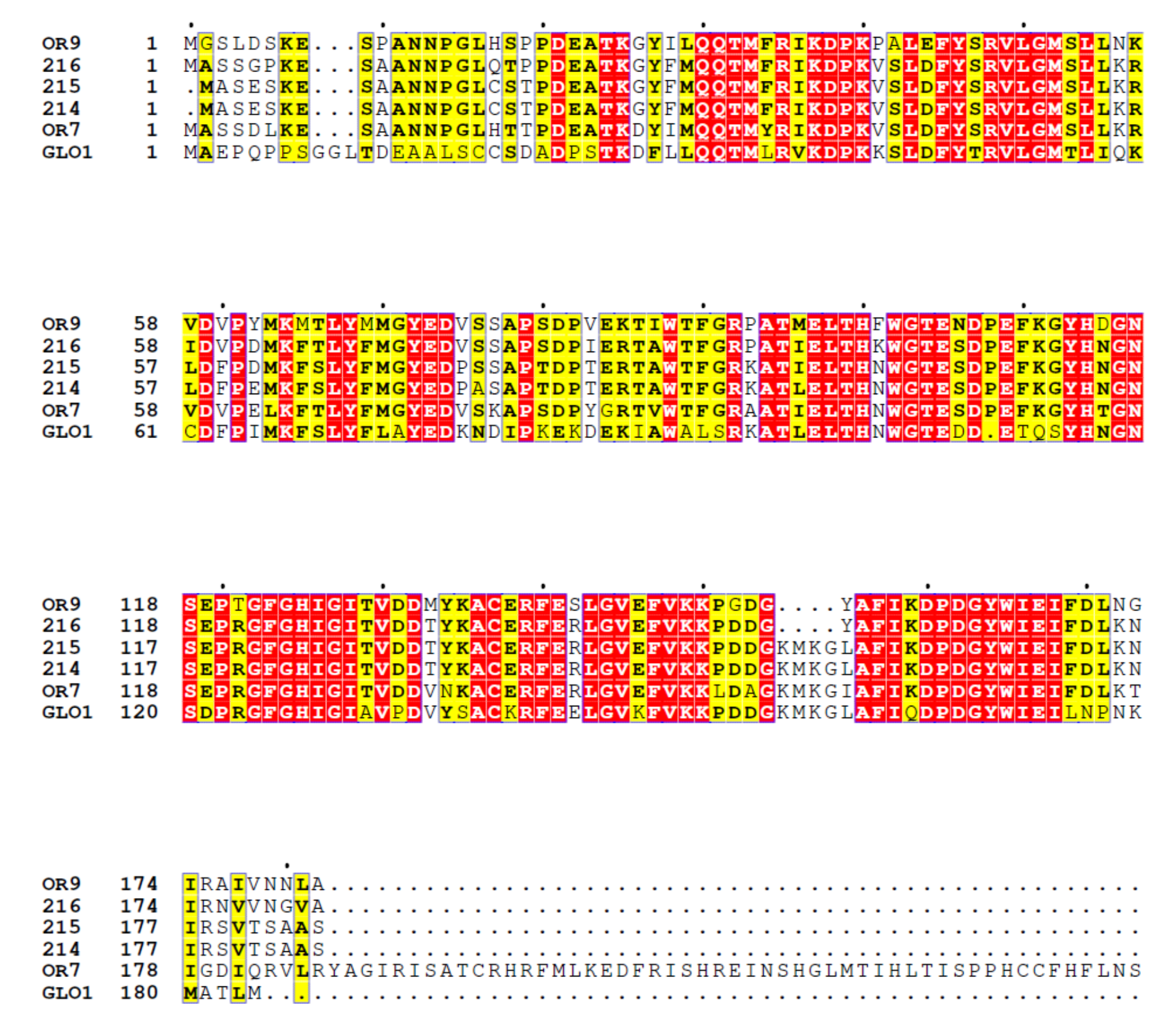
**

**Supplementary Figure 5.** **Multiple sequence alignment of OR9 and its ancestors**. Multiple sequence alignment of OR9 and its predicted ancestor sequences was generated using the ESPript3 server after aligning via Clustal omega.


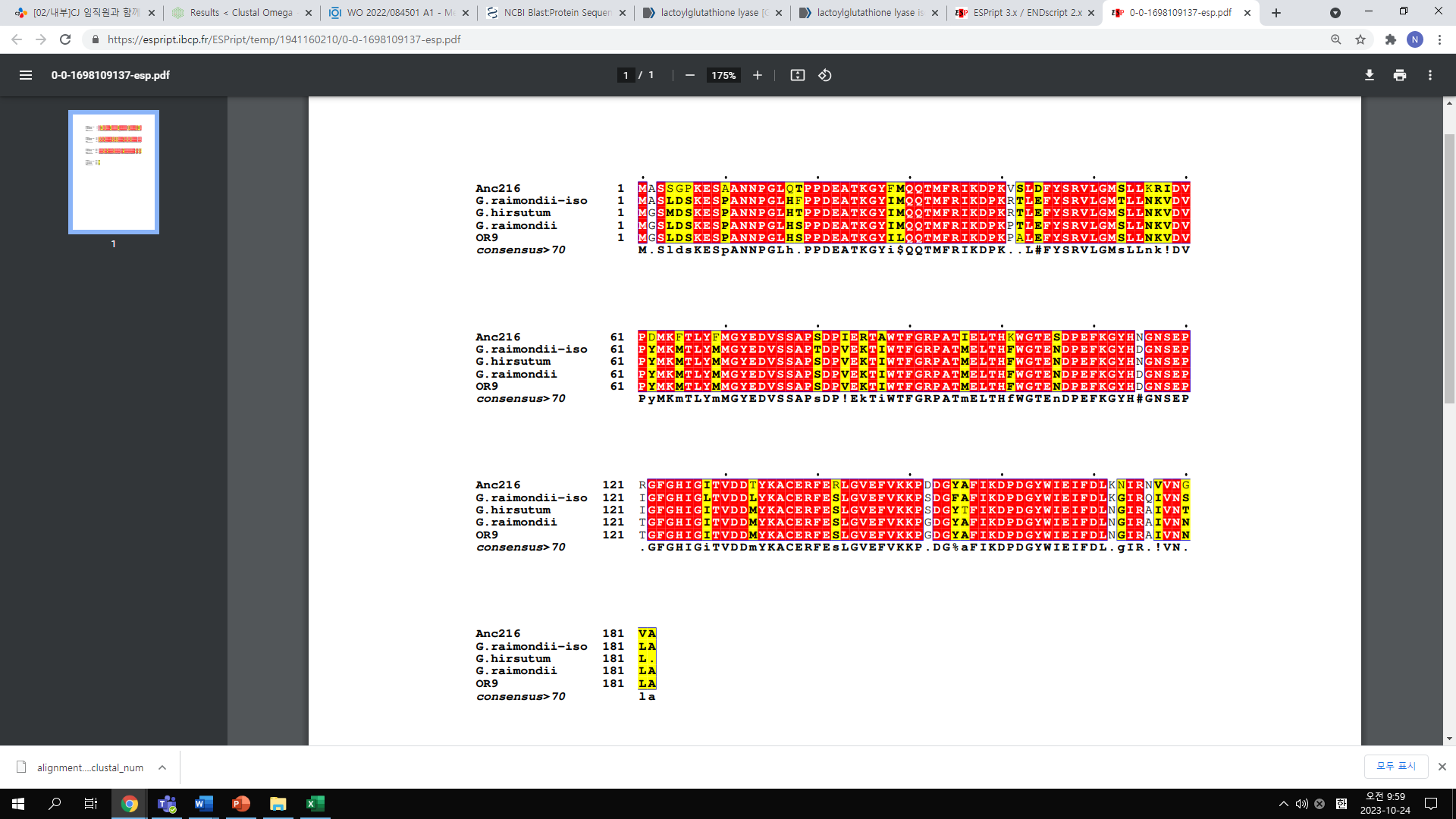


**Supplementary Figure 6.** **Multiple sequence alignment of SPG homologs exhibiting high DON-degradation activity**. Multiple sequence alignment was generated using protein sequences of ancestor 216 and the four SPG homologs validated in-house to exhibit high catalytic activity on DON.

**Supplementary Schemes**

**Supplementary Scheme 1. Enzymatic transformations of deoxynivalenol**. Reported DON degradation pathways via different microorganisms and enzymes.

**Supplementary Data**


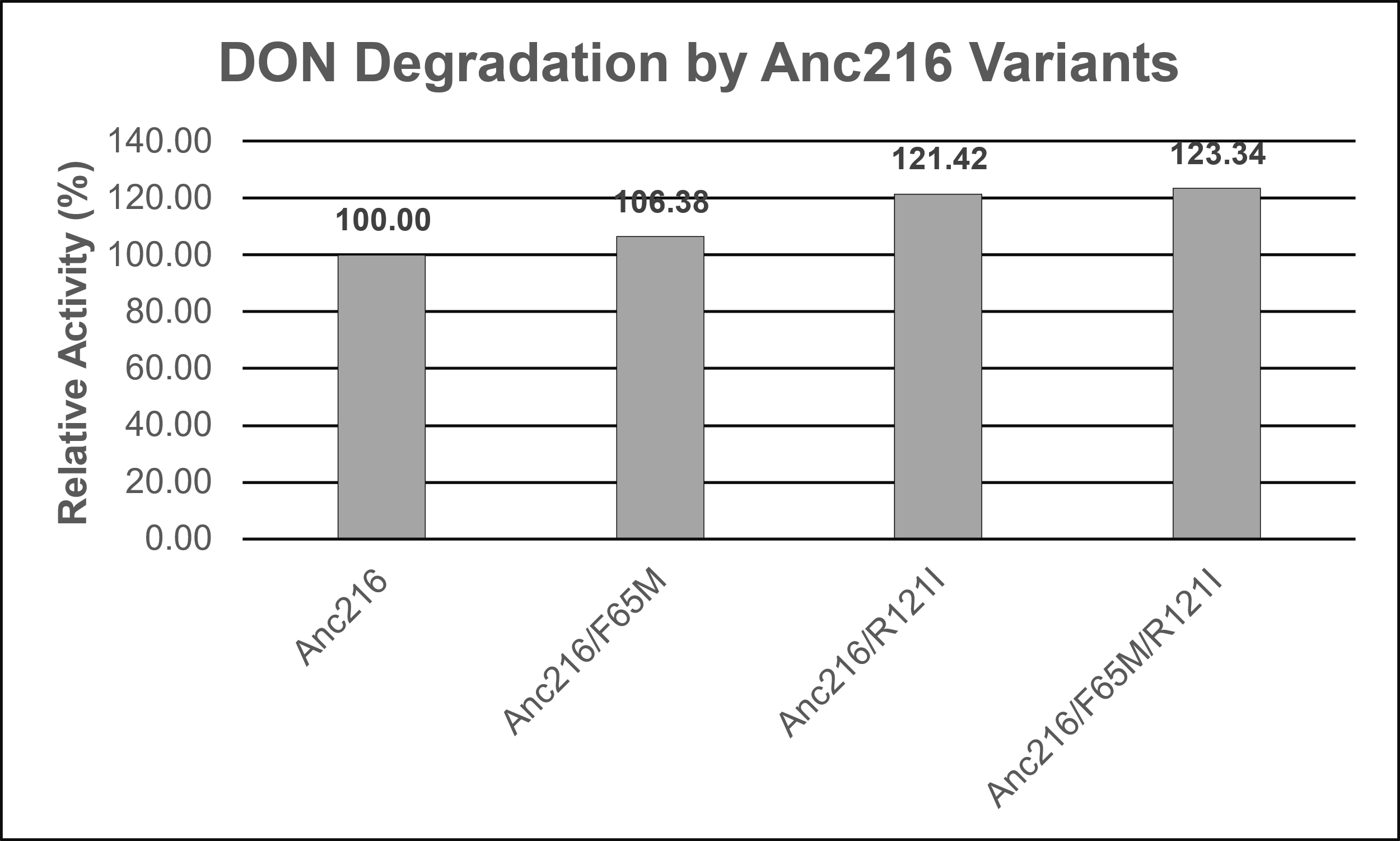


**Supplementary Data 1.** **Relative activities of Anc216 variants**. Relative activities of Anc216 variants (M1/M2) were determined via HPLC-UV analysis.


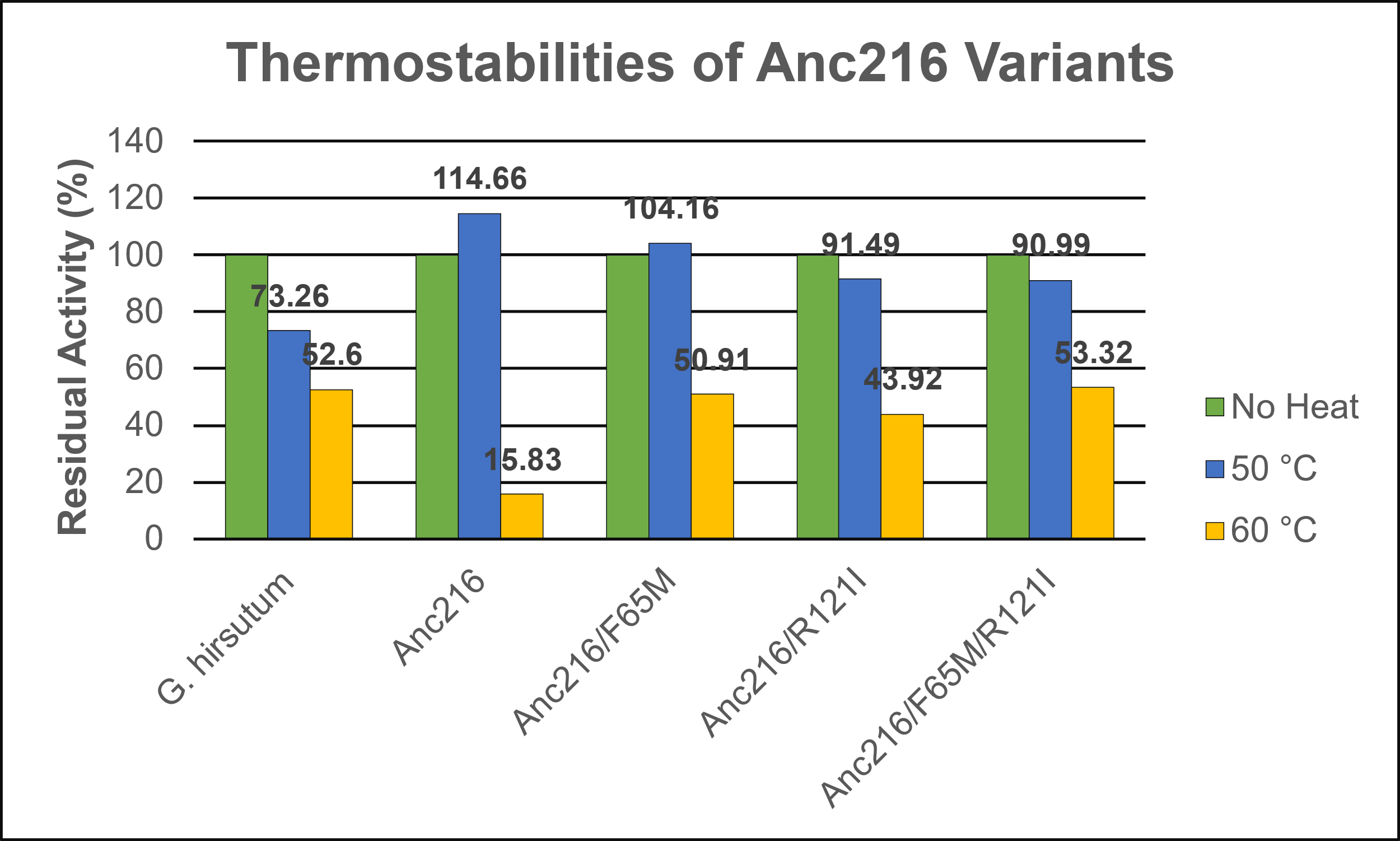


**Supplementary Data 2.** **Thermostabilities of Anc216 variants**. Thermostabilities of Anc216 variants (M1/M2) were determined after pre-incubating each variant at temperatures of 50 oC and 60 oC for 10 min. prior to enzyme assays and HPLC-UV analysis.

**Supplementary Data 3.** **DON degradation activities of the selected mutations from residue conservation analysis.** DON degradation assays were conducted as outlined in Methods section.


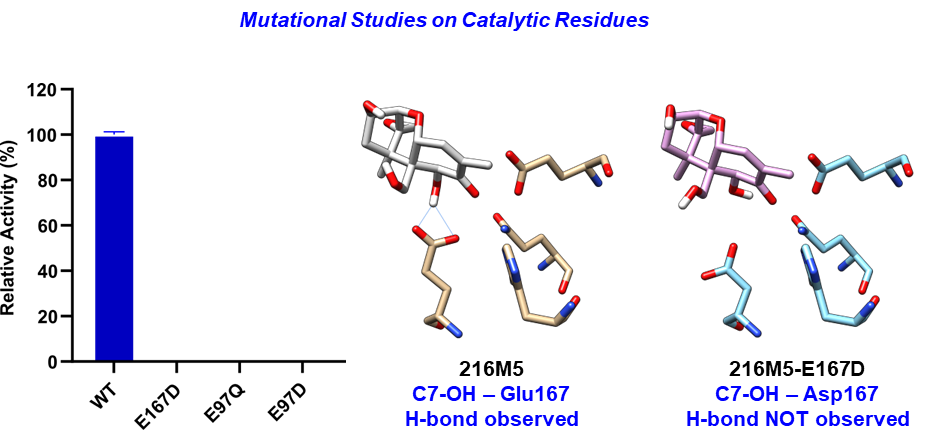


**Supplementary Data 4.** **Mutational studies on the catalytic residues of SPG**. Site-directed mutants of SPG variant Anc216 were generated to analyze the effect of mutating the catalytic glutamate residues E167 and E97. Docking analyses show absence of hydrogen bond when Glu167 is mutated to Asp167.


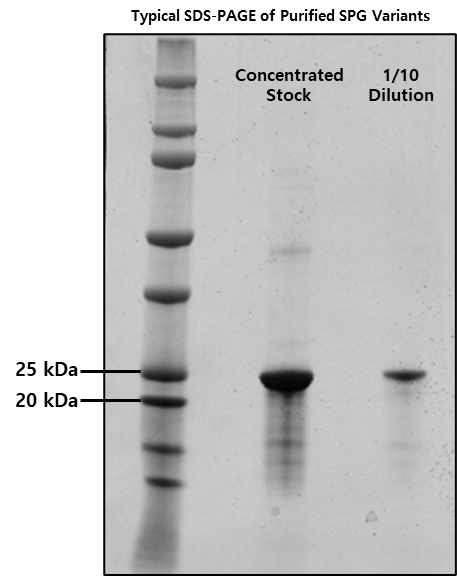

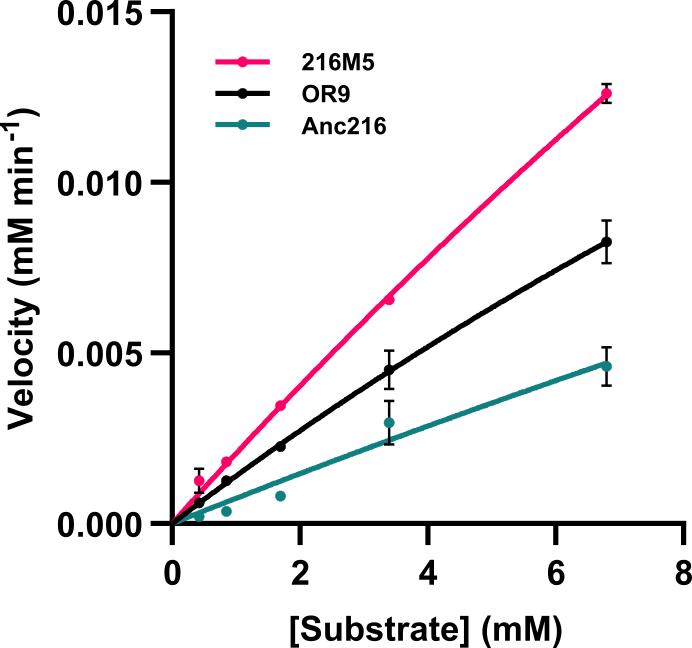


**Supplementary Data 5.** **Typical SDS-PAGE of Purified SPG Variants, and Enzyme kinetics analysis of OR9, Anc216, and 216M5**. Kinetic parameters of the SPG variants were determined in Tris-HCl buffer, pH 9.0, at 50 oC. Substrate concentrations of 0.425, 0.85, 1.7, 3.4, 6.8 mM and 20 μM of enzyme was used for each assay. Amount of DON degraded was measured every 30 min. for 2 hours for each enzyme variant.


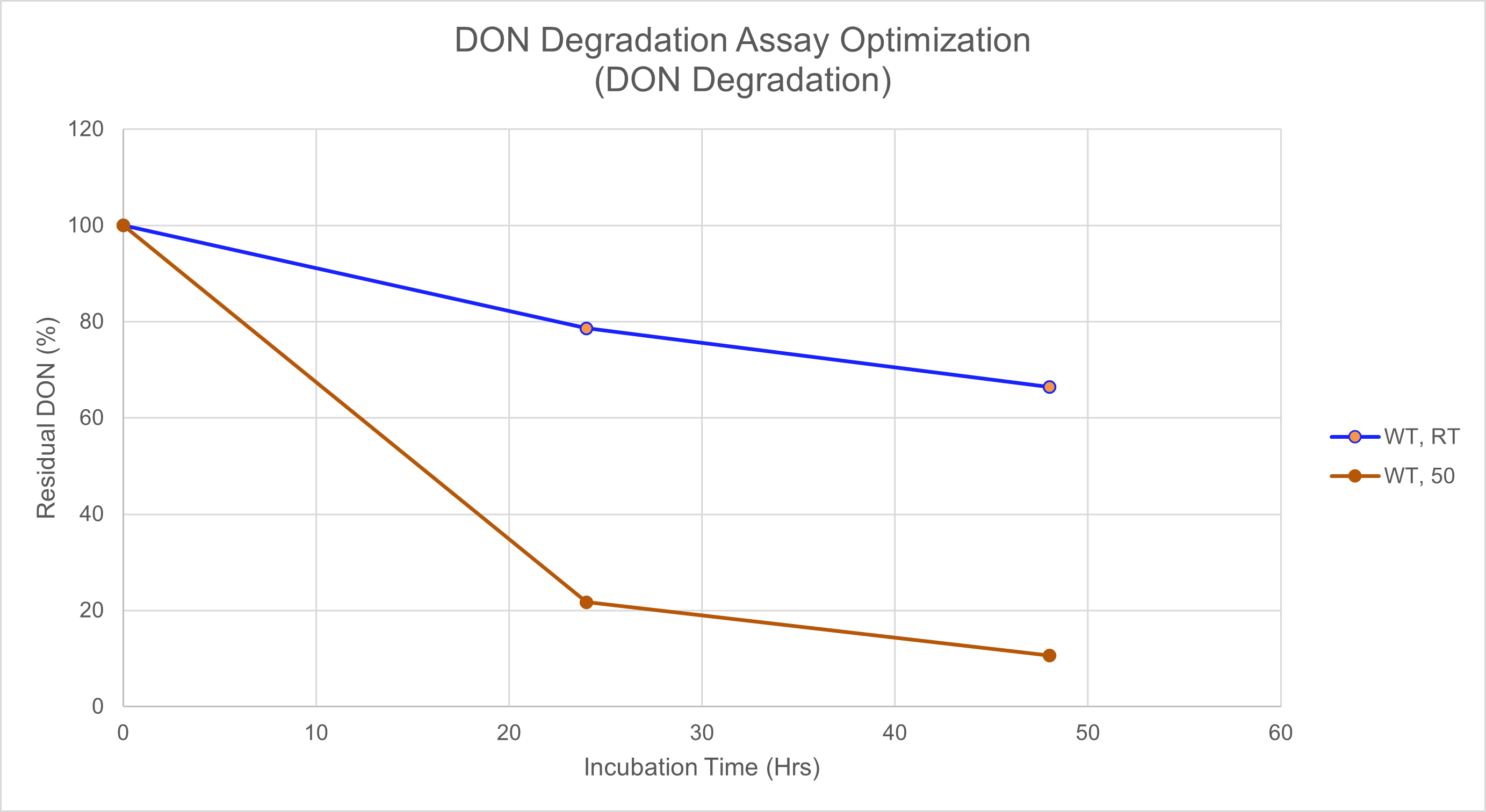


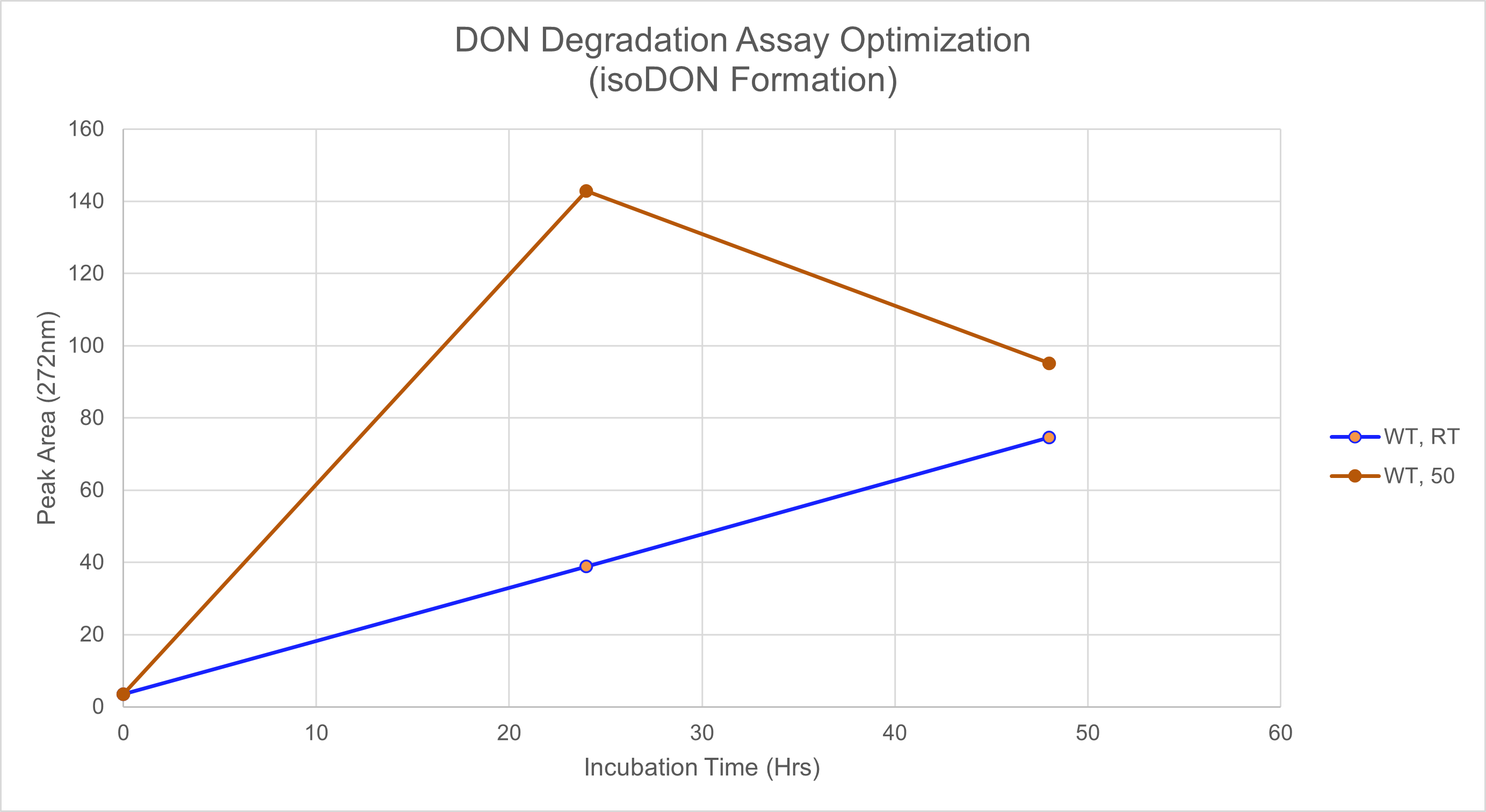


**Supplementary Data 6.** **Spontaneous degradation of isoDON at an elevated temperature**. DON degradation and isoDON production are increased at 50 oC, while spontaneous degradation of isoDON is also accelerated at the elevated temperature.


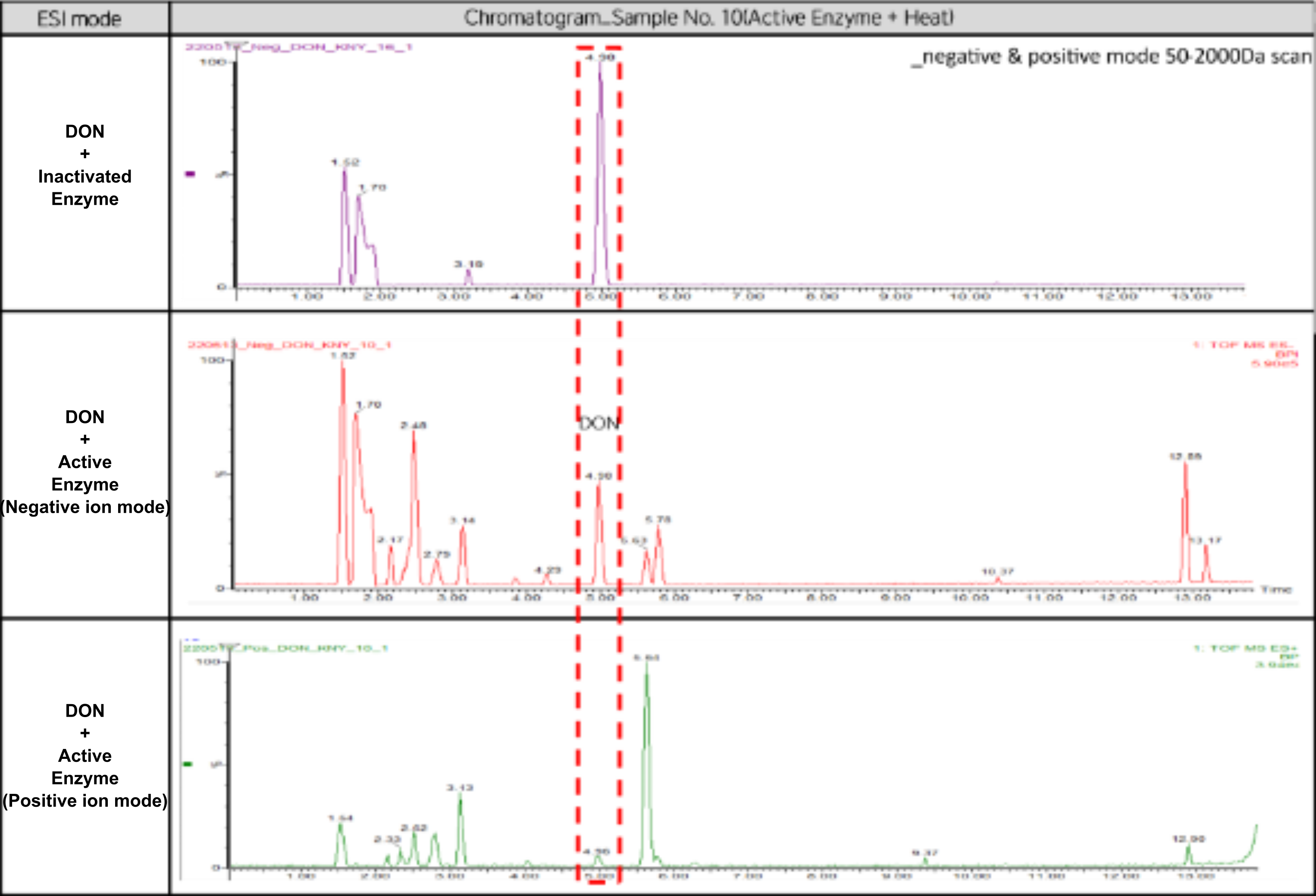


**Supplementary Data 7. LC/MS total ion count (TIC) chromatograms of SPG-DON reaction mixtures.** HPLC analysis shows degradation of DON with concomitant production of unknown degradation products in No. 10 (Active SPG reacted at 50 oC ).


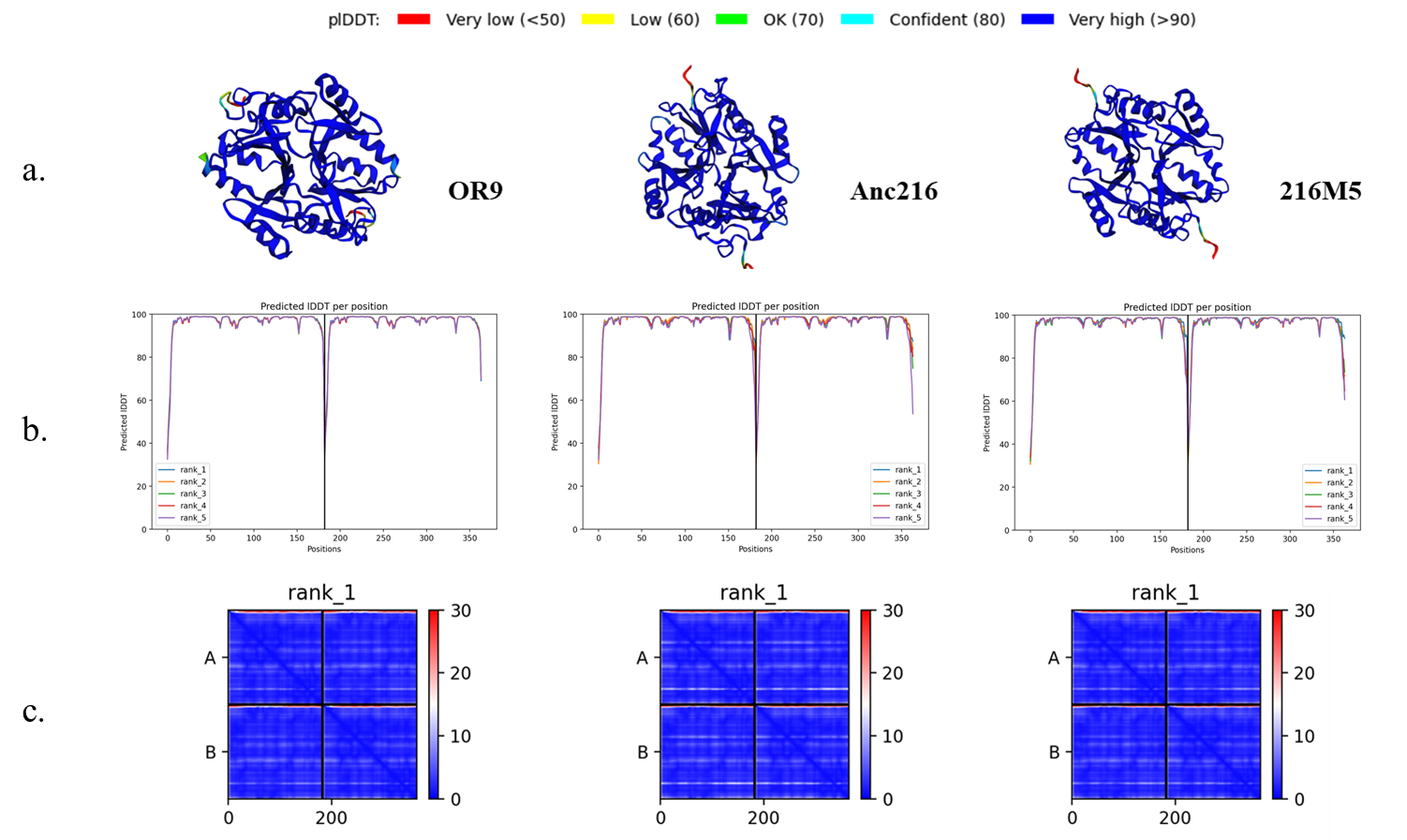


**Supplementary Data 8**. **Estimated model accuracy of the AlphaFold 2-predicted structures of OR9, Anc216, and 216M5.** a. AlphaFold 2-predicted structures (rank 1) of OR9, Anc216, and 216M5, color-coded by the plDDT scores. b. pLDDT score plots for OR9, Anc216, and 216M5. c. Predicted aligned error matrices for rank 1 structures of OR9, Anc216, and 216M5.


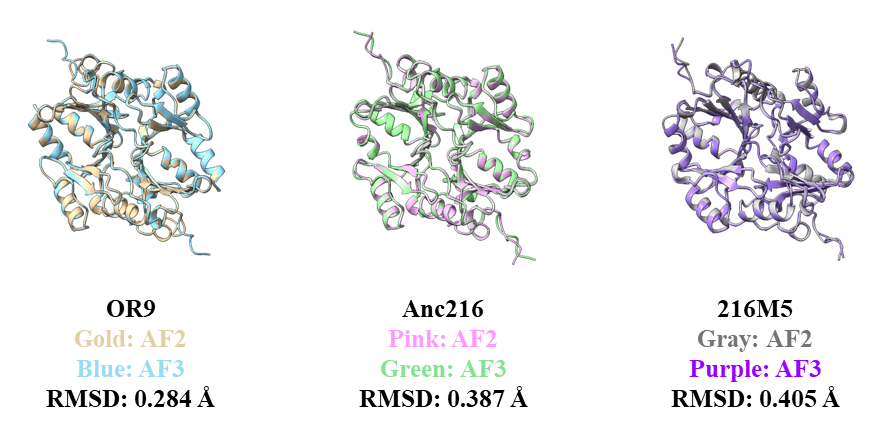


**Supplementary Data 9**. **Comparison of the AlphaFold 2-predicted structures with AlphaFold 3-predicted structures**. Pruned RMSD’s between the AlphaFold 2- and AlphaFold 3-predicted structures indicate that the structures are nearly identical.
